# Persistent conflict in palaeognath phylogeny revealed by quartet-based and ML analyses

**DOI:** 10.1186/s12983-026-00599-1

**Published:** 2026-04-29

**Authors:** Patrick Kück, Alexander Suh

**Affiliations:** Centre for Molecular Biodiversity Research, Leibniz Institute for the Analysis of Biodiversity Change, Adenauerallee 160, 53113 Bonn, NRW Germany

**Keywords:** Palaeognathae, Phylogenomics, Phylogenetic conflict, Ratite paraphyly, Quartet-based inference

## Abstract

**Background:**

The evolutionary relationships among palaeognath birds remain contentious despite extensive phylogenomic analyses. While the ostrich is consistently identified as the earliest diverging lineage, the relationships among non-ostrich palaeognaths remain unresolved, with conflicting topologies and low support across different genomic datasets. In this study, we reanalyzed three genomic marker sets (CNEEs, UCEs, and INTRONs) using alternative maximum-likelihood (ML) approaches and the quartet-based SeaLion method, which assesses phylogenetic signal by evaluating split-pattern information in polarized quartets.

**Results:**

Our results strongly support ostrich as the first split and reinforce ratite paraphyly. However, non-ostrich relationships remain ambiguous, with different best-supported trees emerging across datasets. Among them, the UCEs dataset provided the strongest signal, consistently supporting a ”tinamou-first” scenario with the clade tinamou + moa emerging as sister clade to all other non-ostrich palaeognaths while rhea and kiwi form the most distinct sister-pair. Despite applying quartet-based filtering to reduce phylogenetic noise, substantial conflict persisted, indicating that weak internal branch signal rather than methodological biases alone underlies the lack of resolution.

**Conclusions:**

Our results suggest that the unresolved relationships among non-ostrich palaeognaths are driven by intrinsic data limitations rather than analytical shortcomings. Very short internal branches and extensive incomplete lineage sorting indicate that these divergences may reflect a true polytomy. While this scenario is proposed for Neoaves, a similar process may underlie the persistent uncertainty in palaeognaths. Future progress will depend on expanding comparative frameworks with additional high-quality outgroup genomes, providing a stronger basis for quartet-based phylogenetic inference.

## Background

Palaeognathae is a diverse clade of birds encompassing both flighted and flightless species distributed across multiple continents. This group includes the flighted tinamous (Tinamiformes) of South and Central America, as well as several flightless ratites, such as the African ostrich (Struthioniformes), Australian emu and cassowaries (Casuariiformes), New Zealand kiwi (Apterygiformes), and South American rheas (Rheiformes). Additionally, it comprises the recently extinct New Zealand moa (Dinornithiformes) and Madagascan elephant birds (Aepyornithiformes). Together with the Neognathae, Palaeognathae represents one of the two major lineages of extant birds, forming the class Aves.

For over a century, the phylogenetic relationships among palaeognaths have been a topic of debate, with both morphological and molecular studies yielding conflicting results [1–5]. Traditional classifications distinguished tinamous from the flightless ratites. However, recent molecular studies have challenged this view. Instead, tinamous have been grouped as the sister taxon to the extinct moa nested within traditional ratites [6–9]. Unexpected sister-group relationships such as that between kiwi and the extinct elephant birds have further reshaped our understanding of palaeognath evolution [10–12]. These findings challenge traditional morphological classifications and raise questions regarding the biogeographic history of Gondwana, as well as the role of convergent evolution in the independent loss of flight among ratites [7, 8, 13–15]. As new genomic data continue to emerge, resolving the evolutionary history of palaeognaths remains both complex and ongoing.

The phylogenetic relationships within Palaeognathae remain contentious due to conflicting placements of certain subgroups. For instance, tinamous (along with moa when included) have been variably positioned as the sister group to all non-ostrich palaeognaths, a scenario which we in the following refer to as the “tinamou-first” hypothesis [e.g., 7, 8, 11, 16, 17]. In these studies, either rheas (Rheiformes) [8, 16, 17] or kiwi [7, 11] are inferred as the next lineage to diverge.

Other analyses incorporating an expanded outgroup sampling [13, 18, 19] positioned rheas alongside tinamous, with kiwi forming a distinct sister clade with emus and cassowaries. Additionally, several studies have recovered rheas as the sister group to all non-ostrich palaeognaths [e.g., 6, 13, 10, 20, 12, 21, 17, 22, 9], followed by tinamous [6, 9, 10, 12, 13, 17, 20].

Alternatively, some studies support a “tinamou-last” scenario, where tinamous are placed as the most recently diverging lineage, forming a sister group to emus and cassowaries [21, 22]. Other findings position tinamous within a distinct clade that includes emus, cassowaries, and rheas [18].

**Fig. 1:**
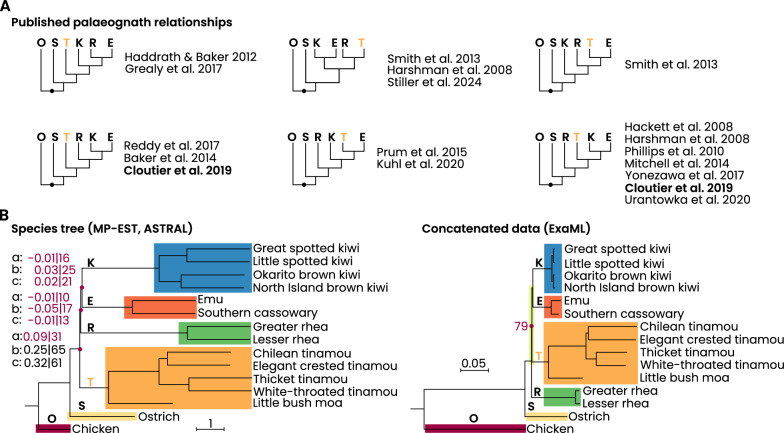
Discrepancies in published palaeognath topologies. Summary of recent phylogenetic hypotheses highlighting alternative relationships among non-ostrich palaeognaths. Rheas (R) are highlighted in green (O: outgroup; S: ostrich; T: tinamous, with moa if included; K: kiwi; E: emu + cassowaries). **A** Representative topologies from previous studies, with the best-supported species-tree and best concatenated ML tree from Cloutier et al. (2019) [17] emphasized. **B** Detailed Cloutier et al. (2019) [17] results from the total evidence dataset (20,850 loci), showing MP-EST and ASTRAL species trees (left) and the concatenated ExaML tree (right). Bootstrap values are shown for nodes with < 100% support. Scale bars indicate branch lengths in coalescent units (MP-EST/ASTRAL) and substitutions/site (ExaML). Terminal branches in species trees are drawn with constant length

The variable placement of tinamous affects the overall topology and underscores the difficulty in confidently resolving deeper splits among non-ostrich palaeognaths, challenges that are exacerbated by short internal branch lengths, rapid radiations, and the decay of phylogenetic signal over time [23–25]. Figure 1 provides a comprehensive overview of the diverse phylogenetic tree results. Using phylogenetic networks and simulations, Suh [26] argued that the initial phase of the super-rapid Neoavian radiation is fundamentally irresolvable due to eight nearly simultaneous speciation events. By analogy, the persistent conflict among palaeognath lineages may likewise stem from very short successive internodes, raising the possibility that their early diversification also approaches a polytomy-like scenario.

Cloutier et al. [17] addressed these challenges by analyzing three distinct genomic marker sets (CNEEs, UCEs, and INTRONs) and retroelements using species tree methods such as MP-EST [27] and ASTRAL [28]. Their analyses recovered a fully resolved topology in which rheas are sister to a clade comprising kiwi and the combined emu-cassowary lineage, with the tinamou-moa clade diverging next after ostriches. This species tree, strongly supported by retroelement insertion patterns and subsampling approaches [e.g., double bootstrapping; 29, 30], contrasts with the alternative ML topology obtained from concatenated data using ExaML [31], where rheas emerge as the sister to all non-ostrich palaeognaths. Cloutier et al. [17] attributed these discrepancies primarily to high levels of incomplete lineage sorting (ILS) [32] across short internal branches, a pattern corroborated by coalescent simulations and gene tree heterogeneity that indicates the presence of an empirical anomaly zone in palaeognaths [33, 34].

Recent divergence-time studies provide additional context for this rapid early diversification. Although we did not estimate node ages ourselves, several fossil-calibrated phylogenomic analyses date the origin of crown Palaeognathae close to the Cretaceous-Paleogene (K-Pg) boundary (ca. 66 Ma), with the major non-ostrich lineages diverging within a relatively narrow time window around this event [12, 19, 35]. Selvatti and Takezaki [35] showed that, across nuclear and mitochondrial datasets and alternative calibration strategies, most estimates converge on a Late Cretaceous to earliest Paleogene age for the palaeognath root, whereas substantially younger Eocene ages are not well supported. Similarly, Yonezawa et al. [12] and the family-level genomic framework of Stiller et al. [19] infer Late Cretaceous-early Paleogene divergences among the main palaeognath clades. This temporal clustering near the K-Pg transition parallels the rapid radiation inferred at the base of Neoaves, and reinforces our interpretation that short internodes, strong gene-tree discordance, and limited phylogenetic signal among non-ostrich palaeognaths are the consequence of a brief burst of diversification rather than solely methodological artefacts.

Two subsequent reanalyses of the same datasets have further explored these issues using different methodological frameworks. Simmons et al. [36] showed that gene-tree misrooting, exacerbated by the use of long-branched neognath outgroups and sparse outgroup sampling, can bias coalescent species-tree inference in palaeognaths, and argued that what was previously interpreted as robust corroboration for a single resolution may instead reflect a shared systematic bias across datasets. Takezaki (2022) [37] compared different sequence types (coding versus non-coding) and demonstrated that loci with high sequence divergence and/or compositional heterogeneity favour alternative placements of kiwi relative to other palaeognaths, underscoring the importance of data type and model adequacy when interpreting palaeognath relationships.

Moreover, node support for internal branch relationships among non-ostrich groups is generally low with only the ostrich consistently emerging as a strongly supported first split, reinforcing the view of a ratite paraphyly while leaving other relationships uncertain (Fig. 1). The presence of short internal branches among non-ostrich clades suggests rapid evolutionary divergence, resulting in only a limited accumulation of phylogenetically informative signal. Consequently, both single-gene and concatenated analyses tend to be less decisive.

Recent large-scale initiatives, such as the B10K family-level phylogenomic project [19], have shown that even dense taxon sampling and genome-scale data often struggle to fully resolve rapid radiations in birds. Indeed, the deepest backbone relationships within Neoaves remain difficult to resolve robustly, despite substantial progress on many shallower nodes [19]. Similarly, Mirarab et al. [38] demonstrated that suppressed recombination regions can introduce systematic biases in Neoavian phylogenies, highlighting how genomic heterogeneity can mislead inference. Together, these studies underline that the persistent uncertainty among non-ostrich palaeognaths may not simply be a matter of insufficient data, but could also reflect intrinsic signal limitations and biases comparable to those observed in Neoaves. This broader perspective stresses the need for approaches that explicitly account for conflicting signals rather than relying solely on increasing dataset size.

Importantly, while Cloutier et al. [17] primarily evaluated ILS effects using single ML gene trees within coalescent frameworks (e.g., ASTRAL-II [28], MP-EST [27]), they also assessed the quality of their concatenated datasets via bootstrap support derived from subsampling approaches such as double bootstrapping [29, 39]. However, as discussed by Cloutier et al. [17], bootstrap values alone are an inadequate measure of confidence in inferred trees, as they may not fully capture underlying model uncertainty and potential data biases [e.g., 40, 41, 30, 42, 43].

One major challenge in reconstructing deep branch divergences is the degradation of phylogenetic signal over time [23]. Rapid radiations further compound this issue by restricting the period available for informative changes to accumulate [24, 25]. In addition, systematic biases such as compositional heterogeneity and long-branch attraction (LBA) often lead to misleading topologies when models fail to account for them adequately [e.g., 23, 44, 45, 46, 47, 48, 49, 50, 51]. To mitigate these biases, Cloutier et al. [17] employed non-coding genomic regions, which are less susceptible to compositional bias than coding sequences [16, 52].

However, additional complementary approaches, such as analyses focused on detecting rate heterogeneity bias in single-clade relationships, identifying signal saturation between clades and within individual species, and rigorously assessing the quality of each concatenated dataset (CNEEs, UCEs, and INTRONs), can further elucidate the sources of tree uncertainty and signal conflict observed by Cloutier et al. [17]. These additional tests not only help differentiate intrinsic data limitations from methodological artifacts but also provide deeper insights into the evolutionary processes shaping palaeognath relationships, ultimately leading to a more comprehensive evaluation of phylogenetic data reliability. Given that traditional bootstrap support can be misleading in large-scale phylogenomic analyses [53], Suh [26] emphasized the importance of assessing reproducibility across independent methods and data types as a more reliable measure of phylogenetic confidence. Following this rationale, complementary analyses such as those described here can provide independent validation of tree topologies, helping to distinguish robust phylogenetic signals from artifacts driven by systematic biases or model misspecification.

Building on these insights, we reanalyzed the three concatenated datasets of Cloutier et al. (2019) [17] using the recently introduced quartet-based method SeaLion [54, 55]. SeaLion evaluates phylogenetic signal by analyzing split-pattern information in polarized quartets (three ingroup taxa plus an outgroup), allowing it to quantify both support and conflict for alternative relationships among predefined clades. Rather than estimating a single global tree, SeaLion focuses on specific questions (e.g., the relative placement of palaeognath subgroups) and provides per-species and per-clade diagnostics of signal quality. Two internal filters downweight quartets with weak or highly conflicting signal and help reduce the influence of systematic biases such as long-branch attraction and convergence, which are known to be problematic in scenarios combining short internal branches with long terminal branches [e.g., 56, 57, 42, 58]. In this way, SeaLion complements coalescent-based and concatenation approaches by highlighting where the data are genuinely informative and where they are dominated by noise or misleading patterns.

**Fig. 2:**
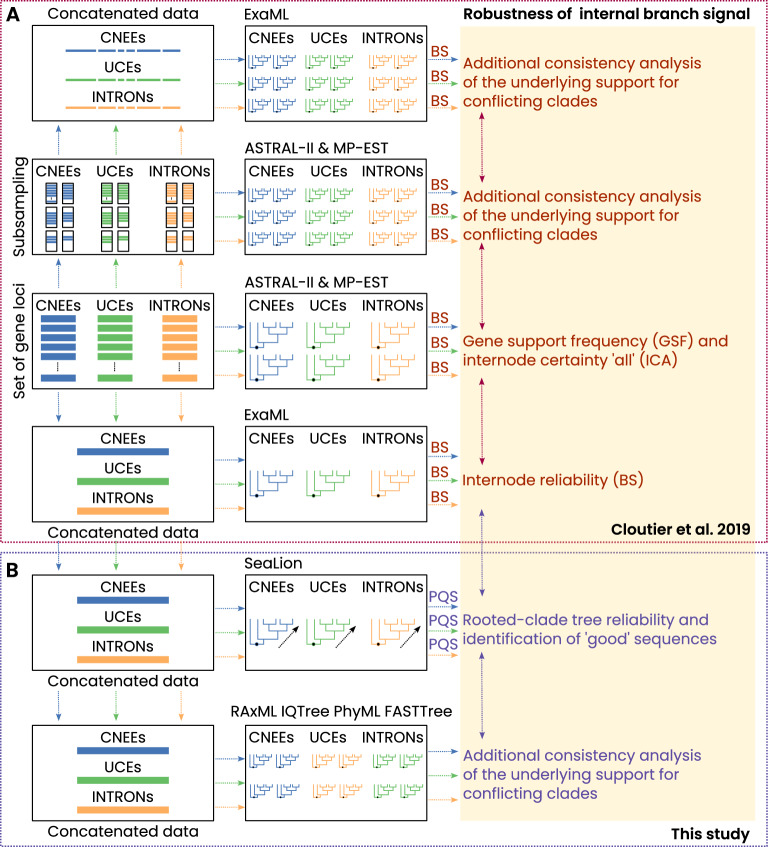
Overview of robustness analyses in Cloutier et al. (2019) and this study. **A** Summary of analyses conducted by Cloutier et al. [17] on CNEEs, UCEs, and INTRONs, including concatenated ML inference (ExaML), single-locus ML gene trees (RAxML), and species-tree estimation (ASTRAL, MP-EST), with internal-branch support assessed using GSF and ICA and subsampling-based bootstrap strategies. **B** Complementary analyses performed here. We reanalyzed the original concatenated datasets with SeaLion and four alternative ML methods. SeaLion evaluates polarized quartet support and applies the RISK and DIST filters to assess signal quality and conflict. ML analyses were used to compare best-tree outcomes across distinct likelihood implementations

To further evaluate the robustness of our SeaLion analyses, we compared the results not only with best trees inferred by Cloutier et al. [17], but also with phylogenetic trees we estimated from the three concatenated datasets using multiple alternative ML methods. For this purpose, each dataset was analyzed using four distinct ML methods (IQ-Tree [59], PhyML [60], RAxML [61] FASTTree [62]), each incorporating different algorithms regarding the estimation of phylogenetic trees. This multifaceted strategy allowed us to assess the sensitivity of ML-based reconstructions to methodological choices and to determine the degree of congruence between the quartet-based SeaLion results, our ML analyses, and the topologies reported by Cloutier et al. [17].

By systematically contrasting the outcome of these approaches, we aimed to pinpoint areas of signal concordance and discrepancy, thereby providing deeper insights into the sources of topological conflict and the overall reliability of palaeognath phylogeny (Fig. 2).

## Methods

### Phylogenomic datasets

We used SeaLion [55] to analyse the three palaeognath datasets (CNEEs, INTRONs, UCEs) from Cloutier et al. [17] to analyse phylogenomic signal for five clade relationships among 14 palaeognath species and a chicken outgroup. For each marker type, SeaLion was applied to the concatenated supermatrix, rather than to individual gene alignments. The three datasets represent different noncoding regions with unique properties, which makes them complementary for understanding evolutionary relationships within palaeognath.

Below is a summary of the main features of each dataset, their differences, and their respective contributions to phylogenetic analysis. Individual gene alignments from each dataset were concatenated into a supermatrix using FASconCAT-G [63]. A summary of key characteristics for each resulting supermatrix is provided in Table 1. For a breakdown of detailed species contributions, refer to Table 2.

CNEEs (Conserved Non-Exonic Elements) are highly conserved regions that are subject to strong evolutionary constraints because they likely perform a functional role (e.g., regulatory regions, conserved genomic functions). This leads to slower evolutionary change in these regions. They were filtered by Cloutier et al. [17] to remove overlaps with coding regions or exons, providing loci free from coding sequence interference. The final dataset consists of 12,676 loci. These loci are relatively stable across species and thus valuable for identifying deeper evolutionary signals.

INTRONs are typically subject to less evolutionary constraint compared to CNEEs (Conserved Non-Exonic Elements), making them valuable for detecting more recent evolutionary signals. This is because introns are noncoding sequences that evolve with less functional constraint, unlike CNEEs, which are subject to stronger selective pressures due to their potential regulatory or functional roles [16, 64]. Intron sequences exhibit greater variability, which provides the resolution necessary for studying phylogenetic relationships at intermediate taxonomic levels. However, this variability can lead to alignment challenges, particularly when comparing intron sequences across divergent species [16, 64]. The final INTRONs dataset of Cloutier et al. (2019) [17] contains 5,016 introns, each derived from a unique gene across the sampled palaeognath species.

Ultra-conserved elements (UCEs) are highly conserved genomic regions whose cores are shared across distantly related taxa, while their flanking regions accumulate substitutions more rapidly and thus provide informative variation at shallower timescales [65]. This combination of a conserved core and more variable flanks makes UCEs particularly useful for resolving both deep and more recent evolutionary relationships. In contrast to the CNEEs, which were explicitly filtered by Cloutier et al. [17] to exclude any overlap with coding regions or exons, the UCE loci in their study were not further masked for exon overlap. As is common for avian UCE datasets, some loci therefore likely include exonic or mixed exonic/intronic sequence.

In this study, we used the subset of 3,158 UCE loci provided by Cloutier et al. [17], which derives from the standard avian UCE marker set described by Faircloth et al. [65] and was assembled across palaeognath and outgroup genomes. We did not re-identify or re-curate UCEs, but instead re-used the published UCE alignments exactly as provided by Cloutier et al. [17] for our quartet-based and ML analyses. No additional filtering was applied to remove potential overlap between UCEs and CNEEs. However, because we analyse CNEEs, INTRONs, and UCEs in three separate concatenated datasets, complete marker independence is not required for our comparisons.

Simmons et al. [36] re-analysed the same UCE matrix from Cloutier et al. [17] and showed that a small subset of loci contains non-orthologous sequence, likely due to paralogy or assembly error. We did not remove such loci here, so any non-orthologous UCEs identified by Simmons et al. [36] remain in our UCE supermatrix. We chose to retain the original locus set to maintain full comparability with Cloutier et al. [17].

**Table 1:** Characteristics of the analyzed supermatrix datasets

Code	Genes	Species	N sites	Indels	Parsimony %	Parsimony sites
CNEEs	12,676	15	4,794,620	00.00%	54.72%	2,623,616
INTRONs	5,016	15	27,890,802	20.48%	21.76%	6,069,039
UCEs	3,158	15	13,293,379	11.15%	12.61%	1,676,295

In this study, we analyzed three supermatrix datasets (CNEEs, INTRONs, UCEs) from Cloutier et al. [17] using the SeaLion quartet framework. The table summarizes key attributes of these datasets, including the dataset code (Code), the number of single genes (Genes), the number of species included (Species), the total alignment length (N sites), the percentage of indel data (Indels), the percentage of parsimony-informative site positions (Parsimony %), and the corresponding number of parsimony-informative sites (Parsimony sites)

### SeaLion processings

#### Clade classification

For each dataset, we assigned the 14 ingroup species and the single outgroup species to six distinct clades to explore internal branch relationships critical for understanding the subgroup phylogeny of Palaeognathae. The five ingroup clades are: emu (E), represented by two species; kiwi (K), with four species; rhea (R), with two species; ostrich (S), represented by a single species; and tinamou (T), comprising five species (Table 2). All three datasets have the same species distribution, maintain identical representation per clade, and consequently involve the same number of analyzed quartets.

**Table 2:** Clade assignment of species

Code	Clade	Species	Common Name	Reference	
E	emu	*Casuarius casuarius*	southern cassowary	Sackton et al. 2018	[66]
E	emu	*Dromaius novaehollandiae*	emu	Sackton et al. 2018	[66]
K	kiwi	*Apteryx hastii*	great spotted kiwi	Sackton et al. 2018	[66]
K	kiwi	*Apteryx mantelli*	north island brown kiwi	Le Duc et al. 2015	[67]
K	kiwi	*Apteryx owenii*	little spotted kiwi	Sackton et al. 2018	[66]
K	kiwi	*Apteryx rowi*	okarito brown kiwi	Sackton et al. 2018	[66]
R	rhea	*Rhea americana*	greater rhea	Sackton et al. 2018	[66]
R	rhea	*Rhea pennata*	lesser rhea	Sackton et al. 2018	[66]
S	ostrich	*Struthio camelus*	ostrich	Zhang et al. 2014	[68]
T	tinamou	*Anomalopteryx didiformis*	little bush moa	Cloutier et al. 2018	[69]
T	tinamou	*Crypturellus cinnamomeus*	thicket tinamou	Sackton et al. 2018	[66]
T	tinamou	*Eudromia elegans*	elegant crested tinamou	Sackton et al. 2018	[66]
T	tinamou	*Nothoprocta perdicaria*	chilean tinamou	Zhang et al. 2014	[68]
T	tinamou	*Tinamous guttatus*	white-throated tinamou	Zhang et al. 2014	[68]
O	outgroup	*Gallus gallus*	chicken	ICGS 2004	[70]

Species are assigned to putative clades with chicken (Gallus gallus) as the sole outgroup (O). The five ingroup clades are defined as follows: emu (clade E) with two species, kiwi (clade K) with four species, rhea (clade R) with two species, and tinamou (clade T) represented by five species (including the little bush moa)

As described avove, molecular evolution varies significantly among our test genes. Some sites evolve slowly, retaining conserved states over long periods. The outgroup plays a key role in rooting topologies and determining branch polarity. It helps distinguish plesiomorphic (ancestral) from derived character states. Phylogenies within individual clades are not analyzed here, as they require separate, focused studies.

#### Definition of clade-quartets and species-quartets

In a SeaLion analysis, clade-quartets and species-quartets serve distinct but complementary roles. Clade-quartets represent the broad phylogenetic hypotheses being tested, while species-quartets are derived from these clade-quartets by selecting one representative species from each group within the clade-quartet. Species-quartets allow for a more focused analysis of site-patterns, helping to differentiate true phylogenetic signals from noise, including misleading plesiomorphic (ancestral, phylogenetically uninformative) signals. Clade-quartets are systematically constructed by combining defined ingroup clades in all possible configurations with the outgroup [55]. This approach enables the examination of clade-quartet topologies that define the key phylogenetic relationships under study.

In this study, we analyse ten different sets of clade-quartets, with corresponding species-quartets each involving the chicken outgroup (O) and one species from three of the five Palaeognathae ingroup clades. For each of these ten clade-quartet combinations, three possible rooted topologies are evaluated (Table 3).

We form clade quartets by pairing an outgroup with three representative ingroup species, each selected from a different clade, ensuring that every quartet features a unique combination of species. The analyses of clade combinations (Table 3) give us data on the relationship between clades.

**Table 3:** Clade-quartet combinations

Quartet	Clade combination				N
EKOR	outgroup	emu	kiwi	rhea	16
EKOS	outgroup	emu	kiwi	ostrich	8
EKOT	outgroup	emu	kiwi	tinamou	40
EORS	outgroup	emu	rhea	ostrich	4
EORT	outgroup	emu	rhea	tinamou	20
EOST	outgroup	emu	ostrich	tinamou	10
KORS	outgroup	kiwi	rhea	otsrich	8
KORT	outgroup	kiwi	rhea	tinamou	40
KOST	outgroup	kiwi	ostrich	tinamou	20
ORST	outgroup	rhea	ostrich	tinamou	10

Clades are organized into unique quartet combinations, with chicken (*Gallus gallus*) designated as the sole outgroup (O). Each clade-quartet comprises the outgroup combined with all unique triplets of ingroup clades, resulting in ten distinct clade-quartets. The total number of species-quartets (N) varies depending on the species available in each clade-quartet. Notably, the tinamou and kiwi clades, which contain the highest number of species (five and four, respectively), yield the greatest number of species-quartets in combinations that include both clades (EKOT, KORT)

#### Calculation of single species-quartet tree support

For each clade-quartet, SeaLion evaluates all possible species-quartet combinations, resulting in three possible rooted topologies per quartet [55]. Each species-quartet is polarized by designating one taxon as the outgroup. Character changes are then interpreted relative to the outgroup state so that derived (apomorphic) states in the ingroup can be distinguished from ancestral (plesiomorphic) ones.

For a given quartet topology *T*(*x*), SeaLion first identifies the number of alignment sites that are compatible with *T*(*x*) and represent shared, derived states among ingroup taxa relative to the outgroup. We refer to this as the apomorphic signal *Na*(*x*), i.e. the number of synapomorphic sites supporting the split implied by *T*(*x*) rather than unique autapomorphies. In parallel, SeaLion uses a ML framework to estimate the amount of convergent or parallel substitution, *Nc*(*x*), that could produce site patterns supporting *T*(*x*) without corresponding to a single shared derived change (i.e. putative convergent signal) [54, 55]. The raw support for topology *T*(*x*) is then defined as

1 $$\begin{aligned} \text {Support for } T(x) = Na(x) - Nc(x) \, . \end{aligned}$$

Thus, high values indicate that the number of synapomorphic (derived, shared) changes supporting *T*(*x*) clearly exceeds the expected amount of convergent signal.

For each species-quartet, the support values of the three possible topologies are finally normalized relative to one another to yield a score between 0 (no support) and 1 (full support) for each topology [54]. This procedure explicitly contrasts phylogenetically informative, derived similarities (synapomorphic signal) with misleading convergent patterns, allowing SeaLion to identify the most likely topology while accounting for potential sources of conflict.

#### RISK and DIST filters

SeaLion applies two complementary filters, DIST and RISK, to identify species-quartets with unreliable or potentially misleading signal before summarizing quartet support at the clade level [54, 55].

First, for each species-quartet, SeaLion computes the support values *S*(*T*) for the three possible topologies $T_1, T_2, T_3$ using Eq. 1. Let $T_{\text {best}}$ and $T_{\text {second}}$ denote the topologies with the highest and second-highest support, respectively. The DIST filter quantifies how clearly $T_{\text {best}}$ is preferred over $T_{\text {second}}$ by calculating the support distance

$$SD_{1,2} = S(T_{\text {best}}) - S(T_{\text {second}}) \,.$$

If $SD_{1,2}$ falls below an automatically optimized threshold ($L_{\text {DIST}}$), the entire species-quartet is discarded for that clade-quartet. Conceptually, DIST removes quartets where the best and second-best trees are nearly tied in support, indicating weak or ambiguous signal rather than a clearly preferred topology.

The RISK filter focuses on the composition of the signal that supports $T_{\text {best}}$. For this topology, SeaLion computes the ratio

$$\text {RISK} = \frac{Nc(T_{\text {best}})}{Na(T_{\text {best}})} \,,$$

where *Na* is the synapomorphic (apomorphic) signal and *Nc* is the ML-estimated convergent signal. Low RISK values indicate that derived, shared characters dominate, whereas values approaching one indicate that convergent or noisy patterns contribute strongly to the apparent support. Quartets with RISK exceeding a second threshold ($L_{\text {RISK}}$) are excluded, because even the best topology for that quartet is judged to be at high risk of being misled by convergence or long-branch effects [55].

In practice, a species-quartet is retained only if it passes both filters (i.e. if $SD_{1,2} \ge L_{\text {DIST}}$ and $\text {RISK} \le L_{\text {RISK}}$). We never replace the best topology with the second-best one. Rather, we discard quartets for which either the relative support is too small (DIST) or the best topology is dominated by convergent signal (RISK). Together, DIST primarily removes quartets with little discriminating power among trees, whereas RISK targets quartets in which long branches and convergence are likely to mislead the inference.

#### Assembly of rooted clade trees

In a final step, SeaLion summarizes the filtered species-quartet scores into a rooted clade tree using a supertree-style aggregation. For the six predefined palaeognath clades, SeaLion enumerates all possible rooted binary trees on these clades and, for each candidate topology, identifies the set of quartets whose splits are compatible with its internal branches. Compatibility is defined via a maximum-biclique criterion on the bipartite graph of clades and supporting quartets [55]. The total support of a candidate tree is then obtained by summing the support scores of all compatible quartets. Because the number of possible rooted binary topologies on six clades is modest, SeaLion can evaluate every tree exhaustively. The best-supported rooted clade tree is therefore obtained through a fully non-heuristic search over topology space, and alternative trees are ranked according to their total compatible quartet support.

#### General SeaLion parameters

For each species-quartet, SeaLion operates on the corresponding subalignment extracted from the concatenated matrix. Only variable sites that are informative for at least one of the three quartet topologies are considered. We required a minimum number of such informative columns per quartet, but for the palaeognath datasets analyzed here all quartets exceeded this threshold. Thus, no quartet was excluded at this preliminary step. The advantage of this design is conceptual rather than practical in this case. Filtering is always performed at the quartet level, so informative sites are never removed globally from the full alignment but only ignored for those specific quartets in which they do not meet the criteria.

To aggregate clade-quartet support from single inferred species-quartet tree signals, we used the median as a more robust central measure than the mean, given its reduced sensitivity to outliers. This enhances reliability when data are skewed or atypically distributed. The median provides a clearer representation of central support and is easier to interpret, particularly with ordinal or categorical data, making it well-suited to our assessment of clade-quartet support [55].

For estimating the expected number of chance similarities that might contribute to false phylogenetic signals (Nc), we applied ML inference as implemented in the P4 package by Foster [71], following the PhyQuart approach of Kück et al. [54]. To identify a reliable phylogenetic signal, the inferred phylogenetically informative characters (Na) must exceed this expected chance similarity count (Na > Nc). Both Na and Nc are calculated for each of the three possible species-quartet trees. We performed P4 analyses under the GTR+$\Gamma$+I model, initializing the shape parameter $\alpha$ at 1 and the invariable sites proportion (I) at 0.3 (indicating 30% expected invariable sites). These parameters were then optimized by P4 for each species-quartet alignment individually.

### ML tree analysis

To complement our SeaLion quartet-based phylogenetic analyses, we performed additional ML tree inferences on the three phylogenomic datasets (CNEEs, INTRONs, UCEs) using four widely employed ML methods: IQ-Tree v1.6.9 [59], PhyML v3.3.3 [60], RAxML v8.2.11 [61], and FASTTree [62]. All ML analyses were conducted under the GTR+$\Gamma$+I model of sequence evolution with otherwise default settings. These analyses serve multiple purposes. First, they provide a robustness check for the phylogenetic topologies inferred by SeaLion by comparing them with the outcomes of full-tree ML approaches. This cross-validation helps to assess the consistency of tree topologies across different analytical frameworks and computational methods.

Furthermore, ML tree inference allows us to incorporate and analyze branch lengths, which are not available in the final rooted-clade trees generated by SeaLion. Branch lengths provide critical information about the evolutionary distance and substitution rates between lineages, offering additional insights into divergence times and relative rates of molecular evolution. By comparing branch lengths across the ML trees from different methods, we can evaluate potential biases or disparities in the resolution of these phylogenomic datasets.

Lastly, employing multiple ML methods ensures a comprehensive assessment of potential methodological biases. Different algorithms vary in their optimization strategies, likelihood calculations, and handling of model parameters, which can influence the inferred tree topology and branch lengths. Thus, this multifaceted approach strengthens the overall reliability and interpretability of our phylogenetic inferences.

## Results

### Clade-quartet tree signal in species-quartets

**Fig. 3:**
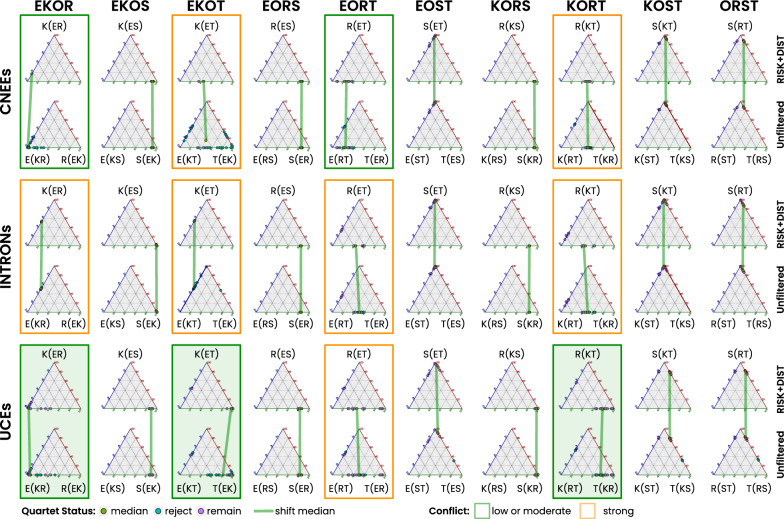
Support for each of the three possible trees of a clade-quartet. Triangle graphs illustrate support for the three possible trees within each clade-quartet. Each corner represents one topology, and each species-quartet is shown as a dot. Dots closer to a corner indicate stronger support for that tree, whereas dots near the center indicate conflict among all three topologies. Dots located along the middle of an edge reflect strong conflict between the two trees connected by that edge. Species-quartet support is shown for each clade combination (top) and dataset (left), comparing unfiltered and RISK+DIST filtered analyses (right). Rejected quartets are shown in blue in the unfiltered graphs, while retained quartets are shown in pink. Green dots indicate the median species-quartet support per topology used for the clade-quartet summary, and green lines connect medians between unfiltered and filtered analyses. For clade combinations including ostrich (S), support consistently favors trees placing ostrich next to the outgroup, and filtering has little effect on median support. For non-ostrich clade-quartets, yellow boxes highlight strong tree-signal conflict, whereas green boxes indicate strong support for the best clade-quartet tree. Shaded green boxes denote cases that favor a tinamou-first scenario

The distribution of single species-quartet related best-trees within each clade-quartet combination closely aligns with the signal strength supporting best single clade-quartet trees. Although the overall impact of the RISK+DIST filter is minimal for the majority of aggregate clade-quartet tree supports, its utility becomes apparent in resolving conflicts among individual best trees across all three datasets. This positive effect is observed for both non-ostrich and ostrich-inclusive clade combinations (Fig. 3 and section 1.1.3 to 1.1.5 of the Additional file 1: Fig. S1).

#### Ostrich-inclusive clade-quartets

In clade-quartets involving the ostrich, SeaLion consistently provides strong support for a topology in which the ostrich (S) forms the first split next to the outgroup ’(O,(S,(*X*,*Y*)))’. This result is robust, with minimal conflict observed in these quartets, suggesting that ostrich holds a well-defined position next to the other Palaeognathae in all three datasets (Fig. 3 and section 1.1.3 of the Additional file 1: Fig. S1). Across all datasets, most species-quartets were retained after RISK and DIST filtering with minimal filtering impact from either RISK or DIST approaches (Table 4 and section 1.1.2 of Additional file 1: Fig. S1). In a small subset of species-quartets within the clade-quartets EOST, KOST, and OSRT, alternative topologies were supported when analyzed without filtering. However, the application of the RISK+DIST filtering approach effectively excluded conflicting species-quartets (see section 1.1.5 of Additional file 1: Fig. S1).

**Table 4:** Number of analyzed species-quartets in each clade-quartet

	CNEEs		INTRONs		UCEs	
Clade-Quartet	u	f	u	f	u	f
**EKOR**	16	$\bigstar$ 1	16	8	16	12
EKOS	8	8	8	8	8	8
**EKOT**	40	$\bigstar$ 3	40	$\bigstar$ 5	40	13
EORS	4	4	4	4	4	4
**EORT**	20	8	20	12	20	13
EOST	10	10	10	10	10	8
KORS	8	8	8	8	8	8
**KORT**	40	$\bigstar$ 7	40	21	40	25
KOST	20	20	20	20	20	16
ORST	10	10	10	10	10	8
Total	176	79	176	106	176	115
Without S	116	$\bigstar$ 19	116	46	116	63

The number of species-quartets analyzed for each clade-quartet across the three datasets, both before filtering (unfiltered: u) and after applying the SeaLion filtering (RISK+DIST: f). Quartets involving ostrich (S) are retained across all datasets, except for tinamou-including quartets in the UCEs dataset (KOST, ORST, EOST), where some quartets are filtered out. The most substantial reductions occur in the four non-ostrich clade-quartets (EKOR, EKOT, EORT, KORT; clade combinations in bold). We denote strongest rejection by $\bigstar$, defined as cases in which $\ge 80\%$ of species-quartets are excluded by filtering relative to the unfiltered baseline ($f/u \le 0.20$). Under this criterion, the CNEEs dataset shows the strongest loss of non-ostrich species-quartets, leaving only 19 quartets in total for these groups, with just one quartet remaining in EKOR and three in EKOT. The number of remaining species-quartets increases from the INTRONs dataset (where EKOT still shows a strong reduction) to the UCEs dataset, where approximately half of the non-ostrich clade-quartets persist (63 out of 116 possible species-quartets)

#### Non-ostrich clade-quartets

In contrast, the non-ostrich clade quartets (EKOR, EKOT, EORT, KORT) are strongly influenced by conflicting tree signals, resulting in significant reductions in the number of species quartets, particularly under RISK filtering (Table 4 and section 1.1.2 of Additional file 1: Fig. S1). The most pronounced rejections occur in the CNEEs dataset. Here, the emu, kiwi, and rhea (EKOR) combination retains only a single quartet, while the emu, tinamou, and kiwi (EKOT) combination retains just three after filtering.

Notably, the optimized RISK thresholds for the majority of non-ostrich clade combinations are close to 1 ($\ge$0.9) or even exactly 1 (e.g., EKOT and KORT in the UCE dataset), indicating an extremely low proportion (close to zero) of apomorphic (Na) signals relative to convergent (Nc) tree signals among species-quartets of the corresponding clade-quartets (for more details see section 1.1.4 of Additional file 1: Fig. S1).

High thresholds indicate the persistence of quartets with strong Nc/Na signal conflicts even after RISK filtering, as the optimization algorithm seeks to balance the proportion of retained and excluded quartets to achieve robust phylogenetic inference. This outcome highlights the challenges of resolving non-ostrich clade relationships, particularly when clade-quartets exhibit a high degree of conflict among species-quartet-analyzed tree signals. Similarly, very low DIST threshold values highlight a high number of species-quartets with only a marginal difference between the best and second-best tree support scores, suggesting that very low tree distances persist even in the filtered datasets.

The support for single clade-quartet trees in the CNEEs, INTRONs, and UCEs datasets varies considerably. Specifically, RISK+DIST filtering generally improves support for tree topologies but does not resolve major conflicts. The best-supported trees across the four non-ostrich clade-quartets (EKOR, EKOT, EORT, and KORT) reveal significant variation in tree topology and potential conflict among the three datasets (CNEEs, INTRONs, and UCEs; Fig. 3 and Additional file 1: File S1). Conflict is strongest in the INTRONs dataset, where support is nearly evenly split between competing topologies across all non-ostrich clade-quartets, followed by the CNEEs dataset, which exhibits pronounced conflict in EKOT and KORT, while the UCEs dataset shows the least conflict, with most clade-quartets converging on a single dominant topology except for localized discordance in EORT.

**Fig. 4:**
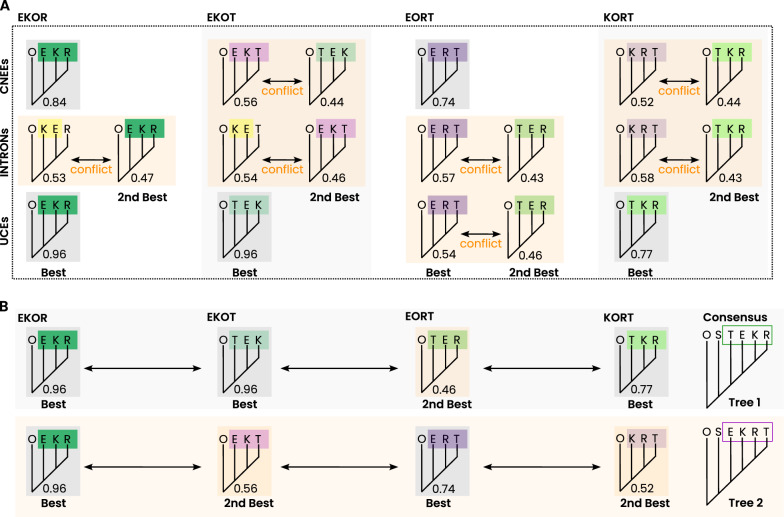
Consistency of best clade-quartet tree support across datasets. **A** Best-supported clade-quartet trees under RISK+DIST filtering (dashed frame) for each dataset (y-axis) and non-ostrich clade-quartet combination (x-axis). Support values range from 0 (no support) to 1 (full support). Gray boxes indicate well supported best-trees for a given dataset and clade-quartet (support $\ge$ 0.7). Where the top two topologies show strong conflict (yellow boxes), the competing second-best topology is additionally indicated. **B** Two overall six-clade hypotheses derived by combining the most consistent clade-quartet signals with selected strongly competing alternatives. Consensus-tree 1 represents a tinamou-first arrangement, whereas consensus-tree 2 represents a tinamou-last arrangement

When assessing overall tree consistency among the most plausible non-ostrich clade-quartet topologies across all three datasets, no single tree is universally supported (Fig. 4 and Section 1.1.7 of Additional file 1: File S1). However, incorporating alternative tree options with support comparable to the best clade-quartet tree reveals a broader phylogenetic structure: one topology, (O,(S,(T,(E,(K,R))))), which is consistent with three of the best trees, favor an early tinamou lineage next to the ostrich (“tinamou-first” scenario). In contrast, another alternative topology, (O,(S,(E,(K,(R,T))))), which positions the tinamou as one of the most recently diverging lineages (a scenario we refer to as the “tinamou-last” hypothesis) is supported by two alternative trees (Fig. 4 and Section 1.1.7 of Additional file 1: File S1).

### Species-specific tree signal contribution

**Fig. 5:**
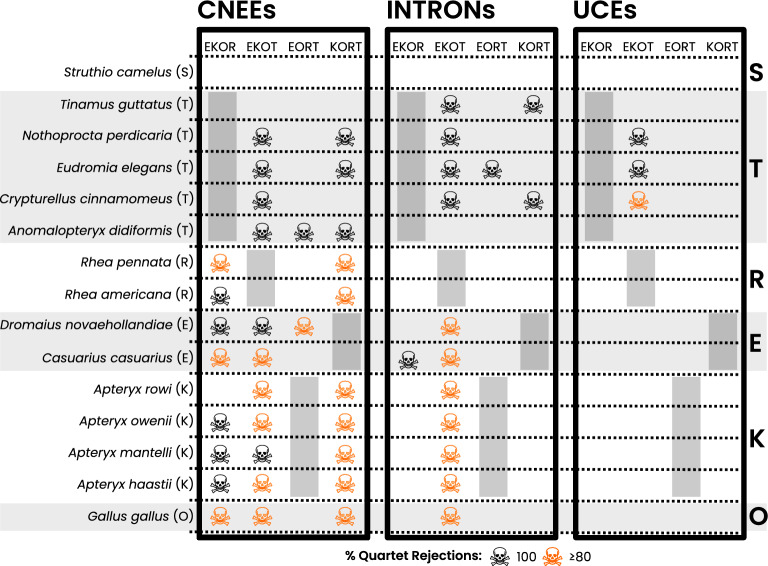
Percentage of quartet rejections relative to total species participation in quartets. The percentage of quartet rejections for each species is displayed across the three datasets, color-coded based on the overall level of rejection: CNEEs (highest rejection rates), INTRONs (moderate rejection), and UCEs (lowest rejection rates). Species are listed on the y-axis, while the x-axis represents the filter approach applied to clade-quartets (RISK+DIST). Species experiencing $\ge$80% quartet rejections are marked with skull symbols: black skulls indicate complete rejection, while red skulls highlight species with at least 80% rejection in clade-specific quartet contributions

Although the rejection of single species-quartets, and consequently the exclusion of species-specific contributions to quartet support, is generally pronounced (Table 4), both the total number of rejected quartets and their distribution among participating species vary significantly across the three datasets (Fig. 5 and section 1.2 of Additional file 1: Fig. S1). The highest levels of quartet rejection occur in the CNEEs dataset, while the UCEs dataset shows the lowest. Rejections are particularly concentrated in the non-ostrich clade-quartets, with tree signal contributions from tinamous being most frequently excluded (Fig. 5). Among these, the clade combination with emus, kiwi, and tinamous as ingroup clades (EKOT) consistently exhibits the highest level of species rejection across all datasets, underscoring its highly conflicted phylogenetic signal.

**Fig. 6:**
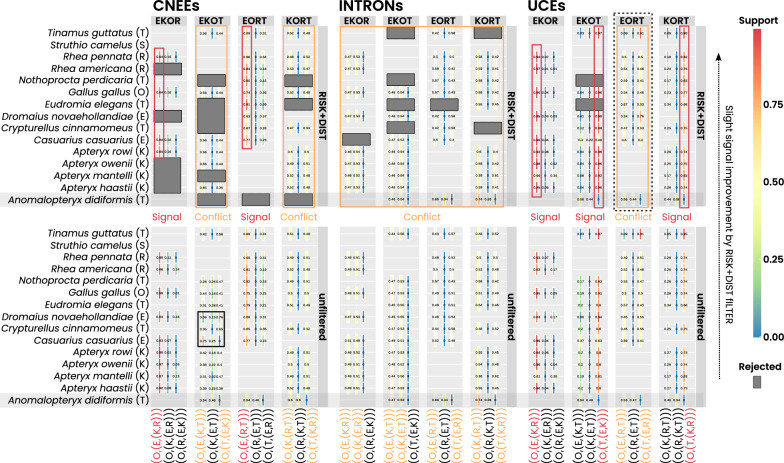
Species support contributions before and after RISK+DIST filtering. Median species-level support (y-axis) for the three alternative trees within each non-ostrich clade-quartet (x-axis, bottom) across the four non-ostrich clade-quartet combinations (x-axis, top). Results are shown for the RISK+DIST filtered data (top) and the unfiltered data (bottom). Colors indicate normalized support strength from 0 (blue) to 1 (red), with intermediate values reflecting conflicting support among alternative trees. Box outlines summarize the dominant pattern per clade-quartet tree (red: strong, consistent signal; yellow: strong conflict). Black boxes mark cases where filtering changes the predominant species-support pattern relative to the unfiltered data; gray boxes indicate clade-quartets with fully rejected species contributions. The Little Bush Moa (*Anomalopteryx didiformis*) is highlighted in gray. For UCEs, EORT shows dataset-specific differences (dashed outline), whereas other clade-quartets predominantly support a tinamou-first arrangement. CNEEs and INTRONs show reduced or conflicted support across several combinations

In each dataset, RISK+DIST filtering resulted in consistent species-level support for individual clade-quartet trees. However, when comparing support patterns for individual clade-quartet trees, the Little Bush Moa (*Anomalopteryx didiformis*) shows distinct support patterns in many non-ostrich clade combinations compared to other species (Fig. 6).

### Final support of rooted-clade trees

**Fig. 7:**
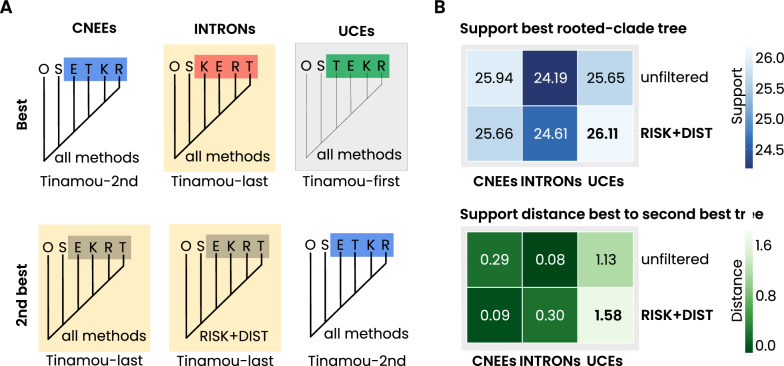
Best and alternative rooted-clade tree support. **A** Best- and second-best supported rooted-clade trees for the five palaeognath ingroup clades ostrich (S), emu (E), kiwi (K), rhea (R), and tinamou (T), inferred from the three concatenated datasets (CNEEs, INTRONs, UCEs) under unfiltered and RISK+DIST-filtered analyses. Trees labeled as ”all methods” indicate topologies recovered in both analyses for the corresponding dataset. **B** Summary of clade-quartet support for the best trees (heatmap) and the corresponding support distances between best and second-best trees. The largest best–second-best distance is observed for the RISK+DIST-filtered UCEs dataset (support difference: 1.58). Yellow boxes mark Tinamou-last arrangements. The gray box marks the Tinamou-first arrangement inferred from the UCEs dataset

Despite the application of quartet filtering to reduce conflict, significant challenges persist in constructing a robust supertree for the five Palaeognathae subgroups. The lack of sufficient support for clade-quartet trees is evident in all final rooted-clade trees (Fig. 7). Across all datasets the ostrich is consistently placed next to the chicken outgroup, but while the UCEs dataset, with the least conflict, weakly supports a “tinamou-first” topology, the more conflicted CNEEs and INTRONs datasets only marginally favor “tinamou-last” scenarios with subtle differences in the placement of emu, kiwi, and tinamou. These discrepancies underscore again the well-documented challenges in resolving the phylogeny of Palaeognathae, even after applying filtering steps designed to improve quartet support (Fig. 7).

### ML tree comparison

**Fig. 8:**
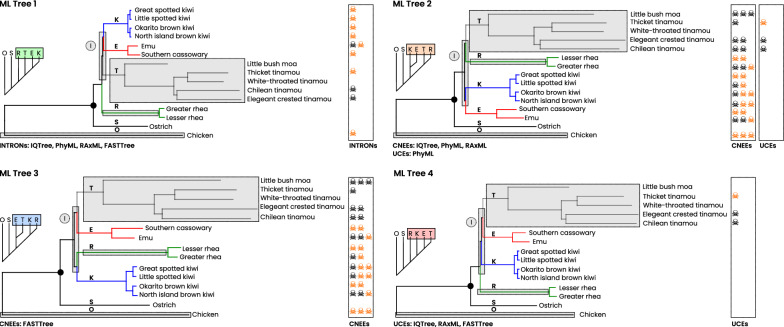
Tree shape and branch-length conditions of best ML inferences. Four alternative ML methods (RAxML [61], IQTree [59], FASTTree [62], PhyML [60]) applied to the CNEEs, INTRONs, and UCEs datasets recovered four recurring palaeognath topologies (Trees 1–4). All trees place ostrich (S) as the first ingroup split after the outgroup (O). Root-to-tip branch lengths are shown for each clade. Notably long branches occur in tinamous (T) and rheas (R), and a long outgroup branch is present. Very short internal branches (I) connect the four non-ostrich clades across all topologies. Symbols mark species frequently excluded by SeaLion RISK+DIST filtering in non-ostrich clade-quartet analyses: black skulls denote complete rejection, and yellow skulls denote partial rejection

To complement the SeaLion analyses, we also conducted ML analyses using RAxML [61], IQTree [59], FASTTree [62], and PhyML [60] on each dataset. These ML analyses produced varying topologies both across and within datasets (Fig. 8 and section 1.3 of Additional file 1: Fig. S1). Notably, none of the ML trees matched the best-supported trees derived from SeaLion or the species-tree from the original study of Cloutier et al. [17].

#### Branch lengths heterogeneity in Best ML trees

Across all datasets, ML methods yield trees with distinct branch length patterns: long internal branches separate the main clades, while the connecting backbone branches are very short, indicating rapid divergence. The tinamou consistently shows the longest branches, with the rhea also having an extended ancestral branch, whereas the kiwi and emu have much shorter branch lengths. The long branches of the chicken outgroup and ostrich further highlight the complex evolutionary dynamics within Palaeognathae (Fig. 8).

#### Best ML trees

Our ML analyses produced four distinct trees for Palaeognathae (Fig. 8), with all methods consistently positioning the ostrich adjacent to the chicken outgroup. In the INTRONs dataset, all analyses converge on the topology where a long-branched rhea diverges immediately after the ostrich, followed by a long-branched tinamou, and finally a short-branched kiwi + emu clade (ML tree 1), closely resembling the concatenated results of Cloutier et al. [17]. The CNEEs dataset shows more variability: one common topology (ML tree 2), recovered by RAxML, PhyML, and IQTree, groups the short-branched kiwi and emu as sister clades while the long-branched rhea and tinamou form another pair, whereas FASTTree recovers an alternative topology (ML tree 3) that pairs the short-branched emu with the long-branched tinamou, leaving the short-branched kiwi and long-branched rhea as a separate pair. In the UCEs dataset, although PhyML replicates the CNEEs topology, other methods yield a tree in which the long-branched rhea diverges first after the ostrich, followed by the short-branched kiwi and an emu + tinamou clade, resulting in the only “tinamou-last” scenario. Notably, none of the best ML trees support a “tinamou-first" scenario.

#### Root-to-tip distances of rejected species contributions

A comparison of rejected species’ support contributions in the non-ostrich clade-quartets (EKOR, EKOT, EORT, KORT) with the corresponding root-to-tip branch lengths in the ML trees reveals a clear pattern: species with longer root-to-tip distances, particularly tinamou species, are more frequently excluded by the SeaLion RISK+DIST filter. Notably, the complete rejection of species with shorter root-to-tip distances, such as those in the kiwi clade, appears to be a secondary effect. These rejections occur because these species are part of the same quartets that are filtered due to the presence of species with longer branch lengths (Fig. 8).

#### Quartet support for Best ML trees

**Fig. 9:**
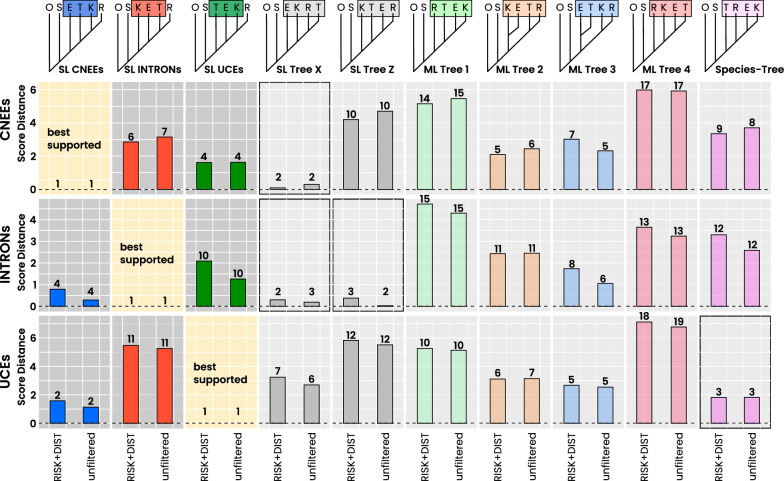
Support distances between best SeaLion trees (SL) and alternative topologies. Support distances (y-axis) between the best-supported rooted-clade tree of each dataset (CNEEs, INTRONs, UCEs) and alternative candidate trees (x-axis top). Results are shown for unfiltered and RISK+DIST-filtered analyses. Numbers above bars indicate the SeaLion rank of the corresponding alternative tree within each dataset. Second-ranked SeaLion alternatives show only minor support differences in CNEEs and INTRONs (SL Tree X and SL Tree Z), whereas larger drops occur in UCEs. All ML-derived alternatives as well as the species tree of Cloutier et al. [17] show larger support distances from the best SeaLion tree

SeaLion consistently assigns lower support to the four ML-estimated rooted-clade trees compared to other topologies (Fig. 9). For example, ML Tree 3 ranks between fifth and seventh across datasets, while ML Tree 2 ranks similarly in the CNEEs and UCEs datasets but falls to eleventh in the INTRONs dataset. In addition, ML Trees 1 and 4 which both place rhea as the first split after the ostrich rank between tenth and nineteenth, showing support values 4 to 6 units lower than the best SeaLion tree. By contrast, the Cloutier et al. (17) species-tree exhibits support only slightly lower than the best UCEs tree, though it is less supported in the other datasets.

## Discussion and conclusions

In this study, we aimed to gain deeper insights into palaeognath relationships by reanalyzing three genomic marker sets (CNEEs, UCEs, and INTRONs) from Cloutier et al. [17] using both conventional ML approaches and the quartet-based SeaLion method [55]. While SeaLion provides useful diagnostics of conflicting signal, our focus here is not on the method itself but on what these complementary approaches reveal about the evolutionary history of palaeognaths.

Across all datasets, our analyses robustly corroborate the ostrich as the earliest diverging palaeognath lineage and reinforce the paraphyly of ratites. This result is consistent with Cloutier et al. [17] and with multiple earlier phylogenomic and multilocus studies that likewise recovered ostrich as the first split within Palaeognathae [e.g., 13, 18, 16, 52]. By contrast, relationships among the remaining non-ostrich lineages remain unresolved. In the coalescent-based species tree of Cloutier et al. [17], rheas were recovered as sister to a clade comprising kiwi and emu + cassowary, with tinamous and moa diverging next. Although our SeaLion analyses also supported a tinamou-first scenario in the UCE dataset, the inferred relationships among the subsequent non-ostrich clades differed markedly, with emus placed next to tinamous rather than rheas. Importantly, the filtering results indicate that the three marker sets differ markedly in their signal-to-noise properties. The pronounced reduction of species-quartets in the CNEEs (and partly the INTRONs) suggests that these partitions may be less informative for non-ostrich relationships, so discordant topologies are not unexpected. In this sense, SeaLion primarily provides a diagnostic of dataset-specific data quality rather than implying that all partitions should converge on a single topology.

The contrasting performance of the three partitions also highlights why datasets with substantial numbers of parsimony-informative sites can nonetheless behave differently in practice. Although CNEEs contain a relatively high proportion of parsimony-informative positions, they represent highly constrained regulatory regions whose evolutionary dynamics may vary across lineages and genomic contexts. Such heterogeneity in selective constraint and rate patterns can increase model violation, amplify discordant signal among loci, and reduce the coherence of quartet support for short, deep internodes. In addition, the short internal branches that characterize early non-ostrich divergences may disproportionately limit the utility of CNEEs if informative changes are sparse or unevenly distributed across loci.

**Fig. 10:**
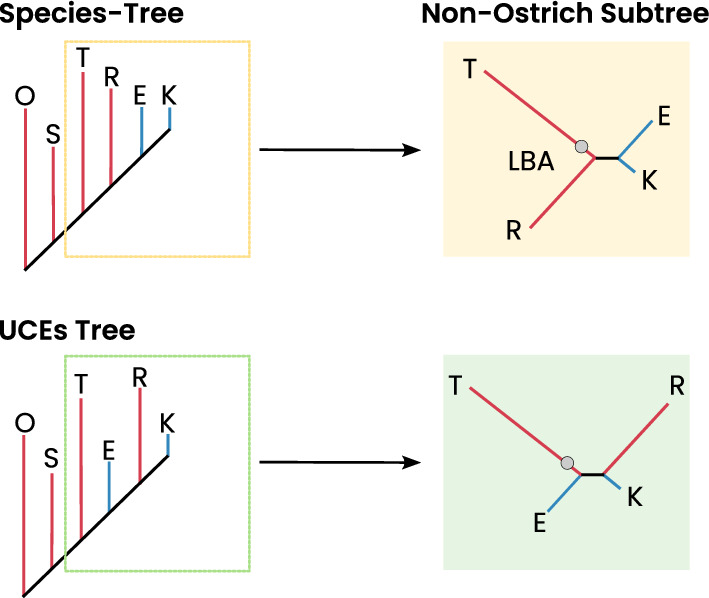
Branch-length arrangement in the Cloutier species-tree versus the best SeaLion UCE tree. Comparison of branch-length distributions mapped onto the coalescent species-tree of Cloutier et al. (2019) [17] (top) and the best-supported SeaLion tree inferred from UCEs (bottom), using branch lengths estimated from our ML analyses. Long branches are indicated in red, short clade branches are indicated in blue. Panels highlight the non-ostrich subtree configuration for visual comparison. In the species-tree, long branches of the ingroup clades are clustered next to the long outgroup branch, while the short branches of the kiwi and emu clades are placed together at the tip of the tree. In contrast, the best-supported SeaLion tree, derived from the UCEs dataset (bottom), shows a mixture of short and long branches along the backbone of the four non-ostrich clades

Our ML analyses further underscored this difficulty. INTRONs and CNEEs datasets mainly produced topologies prone to long-branch attraction, broadly consistent with the alternative ExaML topology reported by Cloutier et al. [17], in which rheas emerge as sister to all non-ostrich palaeognaths. By contrast, the UCE dataset favored a more balanced topology in which rheas split first, followed by kiwi, with emus and tinamous forming the most derived clade. Although this differs from both the SeaLion results and Cloutier’s coalescent-based tree, the overall pattern across ML analyses highlights the sensitivity of concatenation-based approaches to branch length heterogeneity and weak internal signal rather than robust phylogenetic structure (Fig. 10).

We also acknowledge that Cloutier et al. [17] favored coalescent-based species-tree methods for this system and interpreted the discordance with concatenation as a consequence of extensive ILS across very short internodes. SeaLion does not aim to estimate a species tree under the multispecies coalescent. Instead, it provides a complementary, quartet-based assessment of support and conflict in the concatenated matrices, allowing us to localize where signal is weak, contradictory, or potentially shaped by branch-length heterogeneity. The differences we observe among SeaLion best-supported topologies across marker sets do not necessarily imply that coalescent models are inappropriate. Rather, they indicate that even when examined with an alternative, signal-focused framework, the available data still contain limited and heterogeneous information for resolving the non-ostrich backbone. Thus, our SeaLion results are best interpreted as diagnostics that help explain why both concatenation- and MSC-based analyses struggle to converge on a single stable solution for these short, deep splits.

Recent reanalyses of the same Cloutier et al. [17] datasets have further highlighted that data-type specific issues can contribute to the observed discordance. In particular, Simmons et al. [36] showed that a subset of UCE “loci” in the original matrices do not consist of strictly orthologous sequences and that removing such problematic loci can alter support for alternative topologies. In our study, we deliberately reused the published, curated alignments of Cloutier et al. (2019) [17] without additional locus pruning, so our UCE results should be interpreted with this caveat in mind. Nevertheless, it is noteworthy that, even under this conservative re-use, the UCE dataset still exhibits fewer strongly rejected species-quartets and provides the comparatively strongest signal for a tinamou-first scenario. This pattern suggests that, despite reported locus-level issues, the UCEs partition retains substantial phylogenetic information, while also underscoring the importance of future work that systematically evaluates orthology and locus quality across all marker types.

Taken together, these observations are consistent with the general difficulty of resolving short, deep internodes generated by rapid successive divergences. This phenomenon has been described as the “anomaly zone", where gene tree heterogeneity and incomplete lineage sorting (ILS) produce discordant species-tree signals [33, 34]. While Suh [26, 72–74] emphasized such processes in Neoaves, our results suggest that similar dynamics also complicate the resolution of non-ostrich palaeognaths. The very short internal branches we observed across datasets are consistent with this interpretation, supporting the idea that these divergences may represent a polytomy that is difficult, though not necessarily impossible, to resolve.

These persistent conflicts in palaeognath relationships mirror a broader pattern in avian phylogenomics. Even with dense taxon sampling, early radiations remain challenging to resolve. For example, the first phase of the B10K project already highlighted the limits of signal recovery across short internal branches [75], and the most recent B10K species tree framework [38] continues to emphasize the difficulty of disentangling rapid divergences. Similarly, Stiller et al. [19] demonstrated that the backbone of Neoaves may fall within an empirical anomaly zone, suggesting that such irresolvable histories could also underlie the persistent uncertainty among non-ostrich palaeognaths.

Additional work on the Cloutier et al. [17] datasets has reached similar conclusions from different analytical perspectives. Simmons et al. [36] showed that gene-tree misrooting driven by long-branched outgroups and uneven taxon sampling can strongly bias coalescent species-tree inference in palaeognaths, and their updated retroelement analyses likewise point to an ancient anomaly zone. Takezaki [37] further demonstrated that different sequence data types and loci affected by nucleotide composition bias yield alternative placements of kiwi, with only modest relative support among competing topologies. Together with these studies, our results suggest that outgroup choice, branch-length heterogeneity and data-type effects are important contributors to the observed discordance, but that even when these sources of bias are considered, relationships among non-ostrich palaeognaths remain weakly resolved.

Expanded taxon sampling should improve the interpretability of SeaLion’s quartet-based diagnostics in future applications. Increasing the number of outgroup representatives is expected to be particularly beneficial because it would reduce the overrepresentation of a single long-branched reference (here chicken) across all quartets and provide independent polarization tests for the same ingroup splits. Broader sampling within ingroup clades, especially among tinamous, would likewise increase the number of independent species-quartets and help determine whether the observed rejection patterns reflect lineage-specific rate heterogeneity or broader dataset-level limitations. However, given the rapid early diversification inferred for several palaeognath lineages, additional ingroup taxa are unlikely to eliminate the deep, long stem branches separating major clades. Instead, expanded sampling primarily increases the number of informative quartets and can reduce long-branch artefacts without changing the intrinsically short internodes [42, 76].

More generally, results from large-scale avian phylogenomics underscore that genome-scale data do not automatically resolve the deepest splits when rapid radiations, ILS, and heterogeneous evolutionary processes coincide [19, 38, 75]. These studies highlight the importance of methodological pluralism, leveraging both coalescent-based frameworks and novel approaches such as quartet-based filtering, to evaluate the robustness of phylogenetic hypotheses. Our findings for palaeognaths therefore fit into a wider pattern across birds. While genome-scale data provide unprecedented resolution for many nodes, the deepest divergences remain problematic, often reflecting genuine biological complexity rather than purely methodological shortcomings.

Overall, our results suggest that the unresolved nature of non-ostrich relationships is best explained by intrinsic limitations of the genomic data combined with the short internodes characteristic of early palaeognath evolution. While quartet-based filtering can mitigate some misleading signals, it cannot fully overcome the lack of informative variation in these branches. Future work may benefit from integrating the growing availability of high-quality outgroup genomes (e.g., from the Vertebrate Genomes Project), expanding neognath outgroup sampling in line with the concerns raised by Simmons et al. (2022) [36] and Takezaki [37], and applying improved models for heterogeneous sequence evolution. Taken together, these approaches will help clarify whether the ambiguous relationships among palaeognaths ultimately reflect a soft polytomy that can be resolved with better data, or whether they represent a hard polytomy indicative of truly simultaneous divergences.

In summary, our reanalyses reinforce ostrich as the earliest diverging palaeognath lineage while highlighting persistent conflict among the remaining groups. In line with recent work on palaeognaths and Neoaves, these conflicts appear to reflect intrinsic data limitations and rapid early radiations, which together obscure deeper relationships. By combining complementary methods and integrating broader comparative datasets, future work can continue to refine our understanding of early bird evolution, even if some divergences ultimately remain irresolvable.

## Additional file

**Additional file 1**: Additional results available as PDF.

## Data Availability

No datasets were generated or analysed during the current study.
