# Supplementary material for: Persistent conflict in palaeognath phylogeny revealed by quartet-based and ML analyses
Source: Front Zool. 2026 Apr 29;23:21. doi: 10.1186/s12983-026-00599-1 (PMC13182139; doi:10.1186/s12983-026-00599-1)
Supplement: Supplementary file 1 — Additional file 1: Additional results available as PDF. [file 12983_2026_599_MOESM1_ESM.pdf]

Ambiguity in Palaeognath Phylogeny – Supplementary File

Patrick Kück
Alexander Suh

August 25, 2025

Contents

1 Additional Results 2
1.1 Clade-Quartet Tree Signal in Species-Quartets 2
1.2 Species-Specific Contribution to Tree Signal in Clade-Quartets 8
1.3 Maximum-Likelihood (ML) Tree Comparison 12

1 Additional Results

1.1 Clade-Quartet Tree Signal in Species-Quartets

1.1.1 Optimized Thresholds for RISK and DIST Filtering

Table 1: **Optimized Thresholds for RISK and DIST Filtering.** SeaLion dynamically optimized filter thresholds to refine phylogenetic signal accuracy. These thresholds define conflict weights for assessing species-quartet signal quality. Ostrich-inclusive clade combinations in the CNEEs and INTRONs datasets are unaffected by either filter ('—-'), while only two clade combinations (EOST, ORST) in the UCE dataset are impacted. In contrast, non-Ostrich clades (bold) are strongly influenced by RISK and DIST filtering. Notably, the optimized RISK thresholds are close to 1 ($\geq$0.9) or even 1 (EKOT, KORT of the UCEs dataset, highlighted in bold), indicating a very low proportion (close to zero) of apomorphic (Na) signals relative to convergent (Nc) signals among species-quartets of corresponding clade-quartets. Very low DIST threshold values again indicate a high number of species-quartets with a slightly better best-to-second-best tree distance above that threshold, which indicates very low tree distances are still present in the filtered dataset.

	CNEEs		INTRONs		UCEs	
**Clade-Quartet**	**RISK**	**DIST**	**RISK**	**DIST**	**RISK**	**DIST**
**EKOR**	0.92	**—-**	0.70	0.03	0.97	0.03
EKOS	—-	—-	—-	—-	—-	—-
**EKOT**	0.96	0.01	0.97	0.05	**1.00**	0.10
EORS	—-	—-	—-	—-	—-	—-
**EORT**	0.91	0.01	0.70	0.10	0.70	0.08
EOST	—-	—-	—-	—-	0.73	—-
KORS	—-	—-	—-	—-	—-	—-
**KORT**	0.92	0.03	0.73	0.07	**1.00**	0.09
KOST	—-	—-	—-	—-	—-	—-
ORST	—-	—-	—-	—-	0.70	—-

End of table 1

1.1.2 Filtered Number of Species-Quartets

Table 2: **Number of analyzed species-quartets in each clade-quartet with separate filtering results.** This table displays the number of species-quartets analyzed for each clade-quartet across the three datasets, both unfiltered and after applying the SeaLion RISK and DIST filters separately. Quartets involving Ostrich (S) remain largely intact across all datasets, while the non-Ostrich clade-quartets (EKOR, EKOT, EORT, KORT) experience the most substantial reductions, particularly in the CNEEs dataset. The strongest quartet rejections occur under RISK filtering, with only one quartet remaining in EKOR and three in EKOT for CNEEs. UCEs retain the highest number of quartets, with 63 out of 116 remaining in non-Ostrich clade combinations.

	CNEEs				INTRONs				UCEs			
**Clade-Quartet**	**unfiltered**	**RISK+DIST**	**RISK**	**DIST**	**unfiltered**	**RISK+DIST**	**RISK**	**DIST**	**unfiltered**	**RISK+DIST**	**RISK**	**DIST**
**EKOR**	16	★ 1	★ 1	16	16	8	16	8	16	12	12	15
EKOS	8	8	8	8	8	8	8	8	8	8	8	8
**EKOT**	40	★ 3	★ 8	27	40	★ 5	★ 8	24	40	13	23	33
EORS	4	4	4	4	4	4	4	4	4	4	4	4
**EORT**	20	8	8	17	20	12	20	12	20	13	19	14
EOST	10	10	10	10	10	10	10	10	10	8	8	10
KORS	8	8	8	8	8	8	8	8	8	8	8	8
**KORT**	40	★ 7	10	20	40	21	21	26	40	25	25	34
KOST	20	20	20	20	20	20	20	20	20	16	16	20
ORST	10	10	10	10	10	10	10	10	10	8	8	10
Total	176	79	97	140	176	106	125	130	176	115	131	157
Without S	116	★ 19	★ 27	80	116	66	65	70	116	63	79	96

End of table 2

1.1.3 Filtered Tree Signal in Ostrich-Inclusive Clade-Quartets

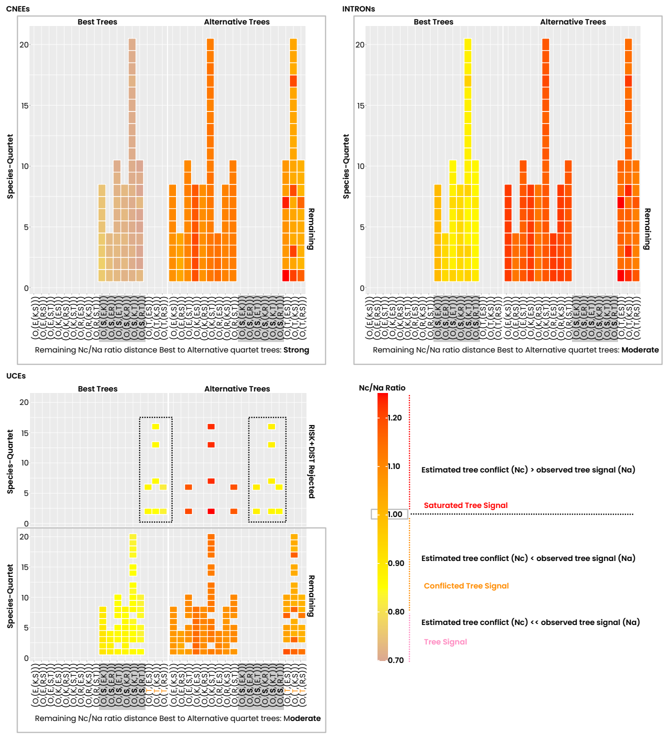

Figure 1: **Heatmap Graphs of Nc/Na Support Ratios for Ostrich-Inclusive Clade Quartets.** Heatmaps illustrating the support values for quartet topologies involving six Ostrich-inclusive (S) clade combinations, with support represented as the ratio of potentially convergent ('Nc') to apomorphic ('Na') character states ('Nc/Na'). Each graph segment corresponds to a dataset (CNEEs: top left; INTRONs: top right; UCEs: bottom left). Quartet topologies examined are depicted beneath each dataset graph, while analysed species-quartets are listed along the y-axis. For each graph, the left segment displays the Nc/Na ratios for species-quartets where the respective topology is the best-supported, while the right segment shows Nc/Na ratios for species-quartets supporting the topology as an alternative (second or third best). The UCEs dataset graph (bottom left) is divided into two segments: the upper section represents species-quartets rejected by RISK+DIST filtering, and the lower section represents non-rejected species-quartets. Across all datasets and species-quartets, Nc/Na ratios are predominantly high, with most quartets yielding values of $\geq$0.8 (yellow to red boxes). This indicates substantial signal conflict between apomorphic and convergently evolved characters in the majority of species-quartets. In the INTRONs and UCEs datasets, best tree-supporting signal (yellow) slightly dominates. Ratios approaching 1 (orange boxes) signify an increasing influence of noise, reducing the likelihood of confidently identifying true tree signal. At an Nc/Na ratio of 1, species-quartet support contributions for the corresponding tree become unreliable. This phenomenon impacts only a subset of best tree signals in the INTRONs dataset. In contrast, best trees derived from the CNEEs dataset exhibit comparably stronger apomorphic tree signals, with most Nc/Na ratios for best trees remaining below 0.8. Ratios $\geq$1 (red boxes) indicate the dominance of conflicting signals over tree-supporting signals, rendering such trees unreliable. This pattern is predominantly observed in alternative trees across all datasets. A distinct apomorphic signal difference between best and alternative trees is clearly visible across all three datasets, with best tree signal consistently identifying the Ostrich clade as adjacent to the outgroup and the remaining two clades as distinct (gray shaded trees). Exceptions occur in a subset of species-quartets with the lowest Nc/Na ratios, where Tinamous are placed next to the outgroup (dashed framed boxes). These quartets exhibit Nc/Na ratios similar to those supporting the Ostrich-outgroup placement, resulting in strong signal conflict. Notably, as shown in the graph, these species-quartets are the only Ostrich-inclusive quartets rejected by the RISK+DIST filter.

1.1.4 Filtered Tree Signal in Non-Ostrich Clade-Quartets

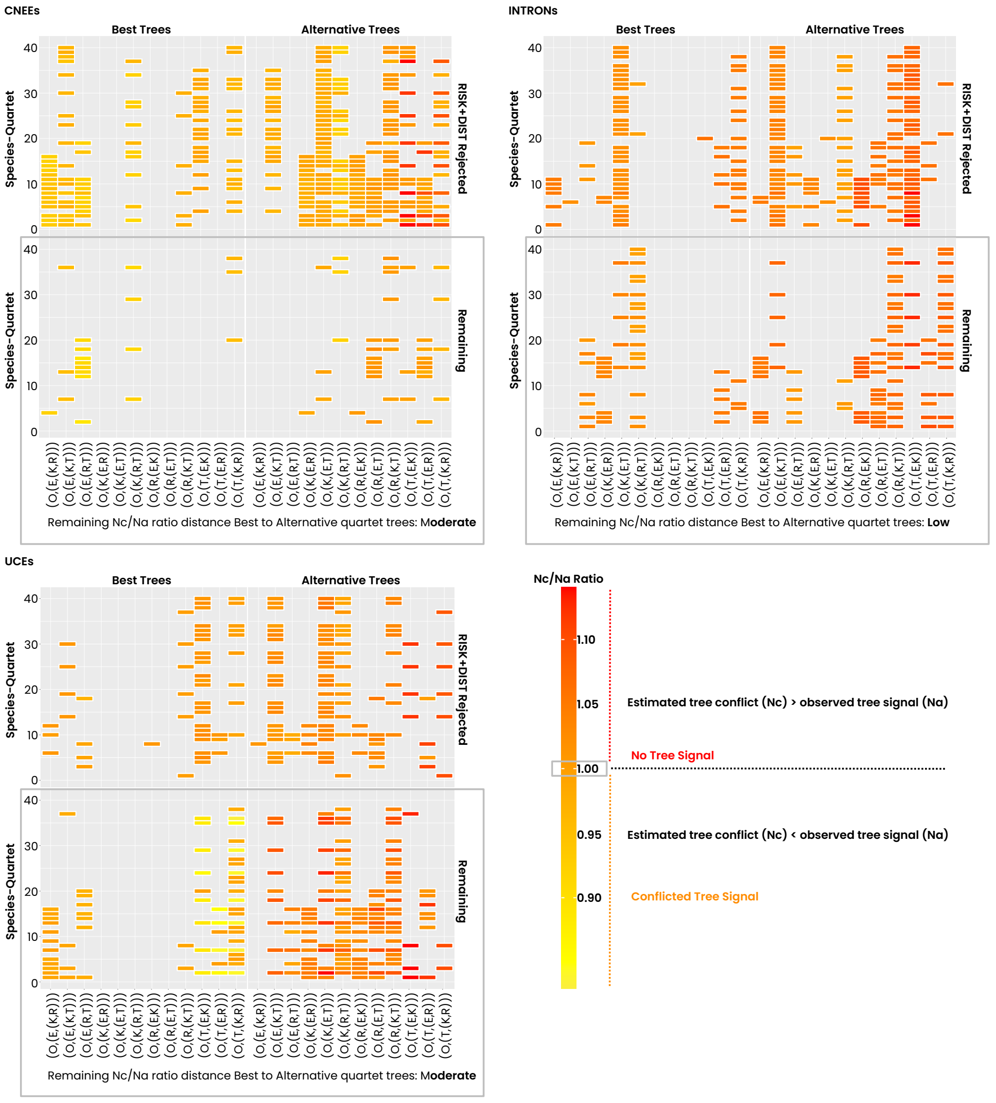

Figure 2: **Heatmap Graphs of Nc/Na Support Ratios for Non-Ostrich Clade Quartets.** Heatmaps illustrating the support values for quartet topologies involving four non-Ostrich clade combinations, with support represented as the ratio of potentially convergent ('Nc') to apomorphic ('Na') character states ('Nc/Na'), as described in Figure 1. While a distinct Nc/Na signal can be observed between best and alternative trees in Ostrich-inclusive clade combinations, the Nc/Na ratios for best trees in non-Ostrich clades are more saturated and closely resemble those of alternative trees. The CNEEs and UCEs datasets show slightly improved Nc/Na ratios for best trees, particularly after RISK+DIST filtering. However, the INTRONs dataset frequently yields highly saturated Nc/Na ratios for best species-quartet trees, indistinguishable from those of alternative trees. Although the RISK+DIST filtering has a marginally positive effect in selecting best trees with improved Nc/Na ratios, it does not overcome the overall pattern of strongly biased tree signals across all species-quartet trees. This underscores the persistent challenge of disentangling true signal from conflict in non-Ostrich clade combinations.

1.1.5 Distribution of Best Species-Quartet Trees in Single Clade-Quartets

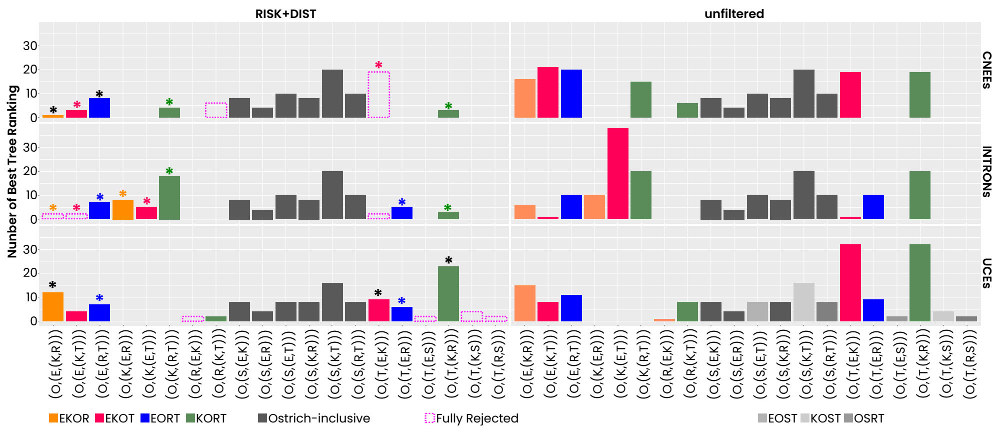

Figure 3: **Frequency of Clade-Quartet Trees as Single Best-Species-Quartet Trees.** The occurrence of single clade-quartet trees (y-axis, left) as best trees (x-axis) in clade-quartet underlying species-quartets is displayed for the three datasets (CNEEs, INTRONs, UCEs; y-axis, right) under unfiltered (right graph) and RISK+DIST filtered (left graph) conditions. Bars are color-coded according to the respective clade-quartet in which the tree is observed. While the number of analyzed species-quartets is significantly reduced following RISK+DIST filtering, some tree conflicts in clade-quartets EKOR, EKOT, and KORT are fully resolved (highlighted by pink dashed lines), resulting in a single overall (median) best-supported clade-quartet tree (marked by a black star). Cases where conflicts among best median supported trees persist are indicated by an orange star. Notably, while some species-quartets in the EKOT clade-quartet of the UCEs dataset still favor alternative best trees, the median support remains robust for the topology placing Tinamou as the first split: (O,(T,(E,K))).

1.1.6 Best Clade-Quartet Trees Across Non-Ostrich Clades

EKOR (Emu, Kiwi, Outgroup, Rhea) In EKOR, the CNEEs dataset consistently supports a close relationship between Kiwi and Rhea with Emu as the first split ((E,(K,R)), even under strong conflict filtering where only a single species-quartet remains. However, the INTRONs dataset favors a different tree ((K,(E,R)), with strong conflict between these two topologies. UCEs, in contrast, supports the (E,(K,R)) tree with more robust evidence. Thus, (E,(K,R)) emerges as the most plausible tree for EKOR when integrating results from all three datasets.

EKOT (Emu, Kiwi, Outgroup, Tinamou) The EKOT clade-quartet shows the most pronounced conflicts. In CNEEs, a best supported but conflicted tree ((E,(K,T)) is favored, with Emu splitting first, while the UCEs dataset supports the alternative ((T,(E,K)), in which Tinamou is not next related to Kiwi, as the best tree. INTRONs strongly conflict between (K,(E,T)) and (E,(K,T)). Considering the strong support in UCEs and the significant conflict within CNEEs, (T,(E,K)) is the most reliable tree for EKOT.

EORT (Emu, Outgroup, Rhea, Tinamou) In EORT, the datasets converge more consistently. The CNEEs and UCEs datasets, along with the RISK+DIST-filtered INTRONs dataset, all support the same topology ((E,(R,T)), with Emu splitting first. This alignment across datasets suggests that (E,(R,T)) is the most reliable tree. Notably, this is the only case where

filtering in the INTRONs dataset significantly influences the outcome, aligning with the other datasets and resolving initial conflicts seen in the unfiltered INTRONs analysis. However, (E,(R,T)) remains in strong conflict with the second-best quartet tree, (T,(E,R)), which suggests that the Tinamou (T) may instead represent the earliest split in INTRONs and UCEs.

KORT (Kiwi, Outgroup, Rhea, Tinamou) KORT presents a highly conflicted scenario. The UCEs dataset strongly supports the (T,(K,R)) topology, with the Tinamou (T) splitting first. In contrast, both the CNEEs and INTRONs datasets favor the alternative tree, (K,(R,T)), where the Kiwi (K) splits first, though this conflicts with (T,(K,R)). Despite this disagreement, the robust support from UCEs for (T,(K,R)), along with its emergence as a similarly well-supported alternative in CNEEs and INTRONs, suggests that (T,(K,R)) may be the most plausible topology for KORT.

1.1.7 Overall Consistency of Best Clade-Quartet Trees

When analyzing overall tree consistency among the four most plausible non-Ostrich clade-quartet topologies across the three datasets, no single tree emerges as globally supported (Fig. 4 of the main pupblication). The most plausible tree in EORT, (E,(R,T)), with the Emu (E) as the first split, directly contradicts the topologies in EKOT and KORT, which support the Tinamou (T) as the earliest split. Additionally, EKOT suggests a more derived position for the Emu, further highlighting the conflicting evolutionary placements across these clade-quartet combinations. Considering the second-best tree in EORT, (T,(E,R)), of the INTRONs and UCEs datasets, which is nearly as well-supported as the best tree, tree consistency for Tinamou as first split improves. Alternatively, disregarding the entire clade-quartet due to its strong signal conflict also enhances overall tree consistency among remaing clade-quartets.

In both scenarios, a broader topology emerges across all four or three clade-quartet combinations, positioning the Tinamou (T) as the earliest split, followed by the Emu (E). The Kiwi (K) and Rhea (R) form the most derived sister clades. This arrangement aligns with the strongly supported placement of the Ostrich (O) near the outgroup, resulting in Consensus-Tree 1: (O,(S,(T,(E,(K,R))))). This tree reflects a cohesive phylogenetic structure where the Ostrich is distinctly positioned next to the other species, supporting a Tinamou-first scenario (Fig. 4 of the main pupblication).

An alternative approach to achieving overall tree consistency involves favoring the strongly conflicted second-best trees in EKOT and KORT over the better-supported best tree from the UCEs dataset. This alternative topology places the Emu (E) as the first split next to the Ostrich and the outgroup, followed by the Tinamou (T), Kiwi (K), and finally the Rhea (R), which pairs with the Tinamou as the most derived clade (Tinamou-last scenario). This arrangement results in Consensus-Tree 2: (O,(S,(E,(K,(R,T))))), aligning fully with the clade-quartet tree composition. However, this tree relies on resolving two significant tree conflicts, undermining its reliability and suggesting it may be less robust compared to the Tinamou-first scenario represented by Consensus-Tree 1 (Fig. 4 of the main pupblication).

1.2 Species-Specific Contribution to Tree Signal in Clade-Quartets

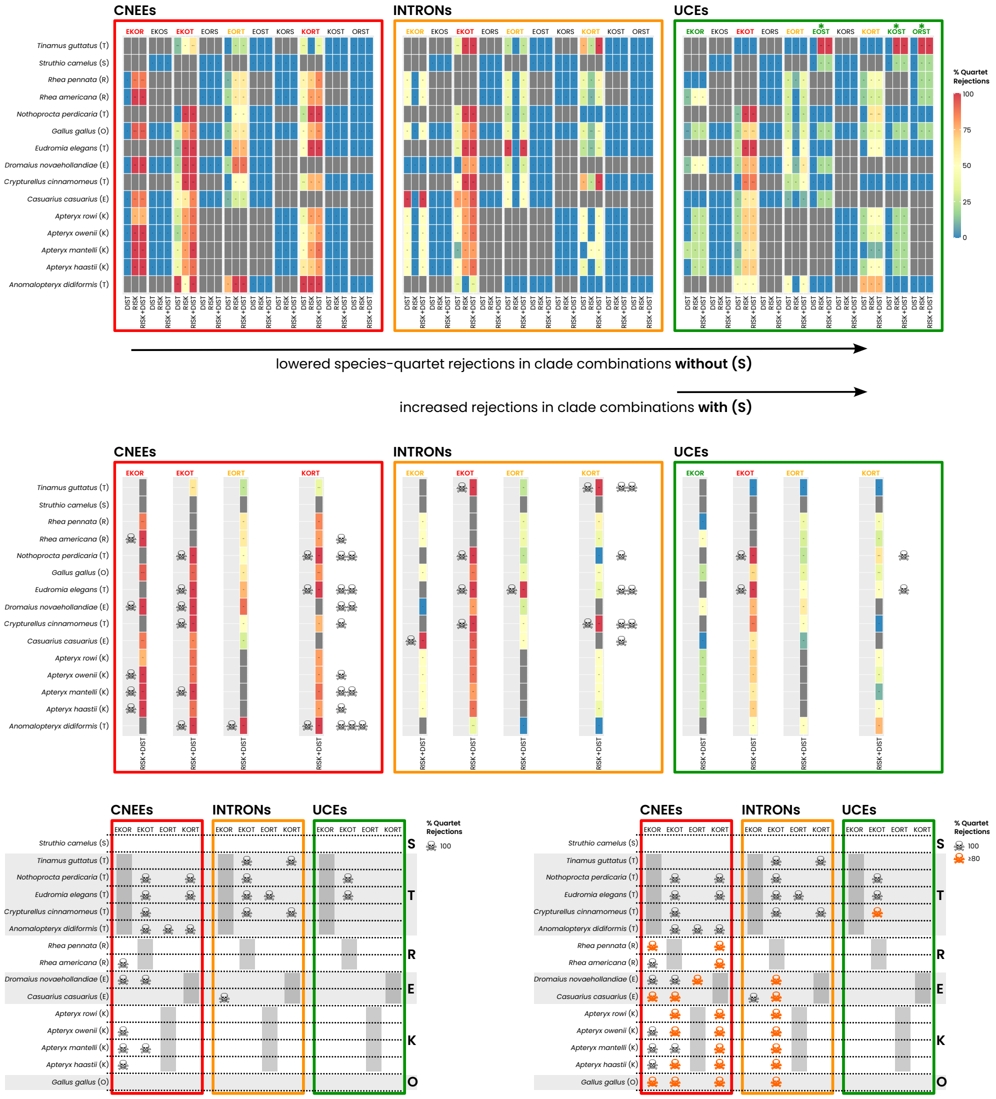

Figure 4: **Percentage of quartet rejections relative to total species participation in quartets.** The percentage of quartet rejections for each species is displayed across the three datasets, color-coded based on the overall level of rejection: CNEEs (red box for the highest rejection rates), INTRONs (yellow box for moderate rejection), and UCEs (green box for the lowest rejection rates). Species are listed on the y-axis, while the x-axis represents the filter approaches applied to clade-quartets (RISK, DIST, RISK+DIST). Rejection percentages are visually coded: dark blue represents 0% rejections, green indicates approximately 25%, yellow denotes around 50%, and red signifies 100% rejections. The top heatmap illustrates rejection percentages across all clade-quartets, while the middle heatmap focuses on the more conflict-prone non-Ostrich clade-quartets. In the lower graphic, species experiencing $\geq$80% quartet rejections under the RISK+DIST filter are marked with skull symbols: black skulls indicate complete rejection, while red skulls highlight species with at least 80% rejection in clade-specific quartet contributions.

CNEEs

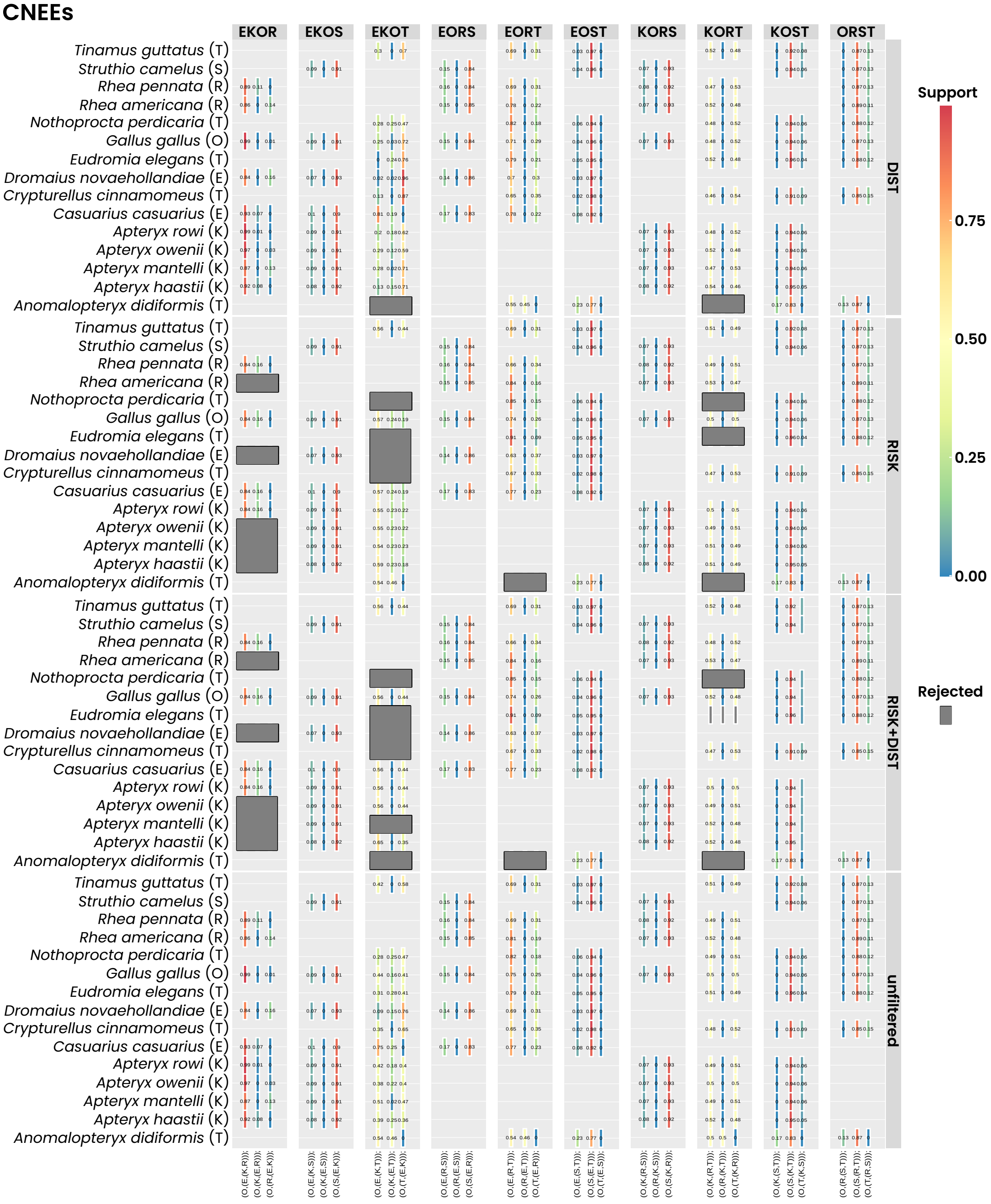

Figure 5: **Species Support Contribution in Unfiltered and Filtered CNEEs (RISK, DIST, and Combined).** The median species-specific support (y-axis, left) for each of the three possible clade-quartet trees (x-axis, bottom) is shown across all ten clade-quartet combinations (x-axis, top) for unfiltered and RISK and/or DIST filtered datasets (y-axis, right). The color scale (legend, right) represents the level of support, ranging from zero (blue) to full support (red), with yellow indicating strong conflict between trees. Clade-quartet trees are highlighted according to the predominance of species support strength: red box frames indicate strong and consistent support within single clade-quartets, while yellow box frames mark conflicted support. Species contributions that were completely rejected in single clade-quartets are highlighted with gray boxes.

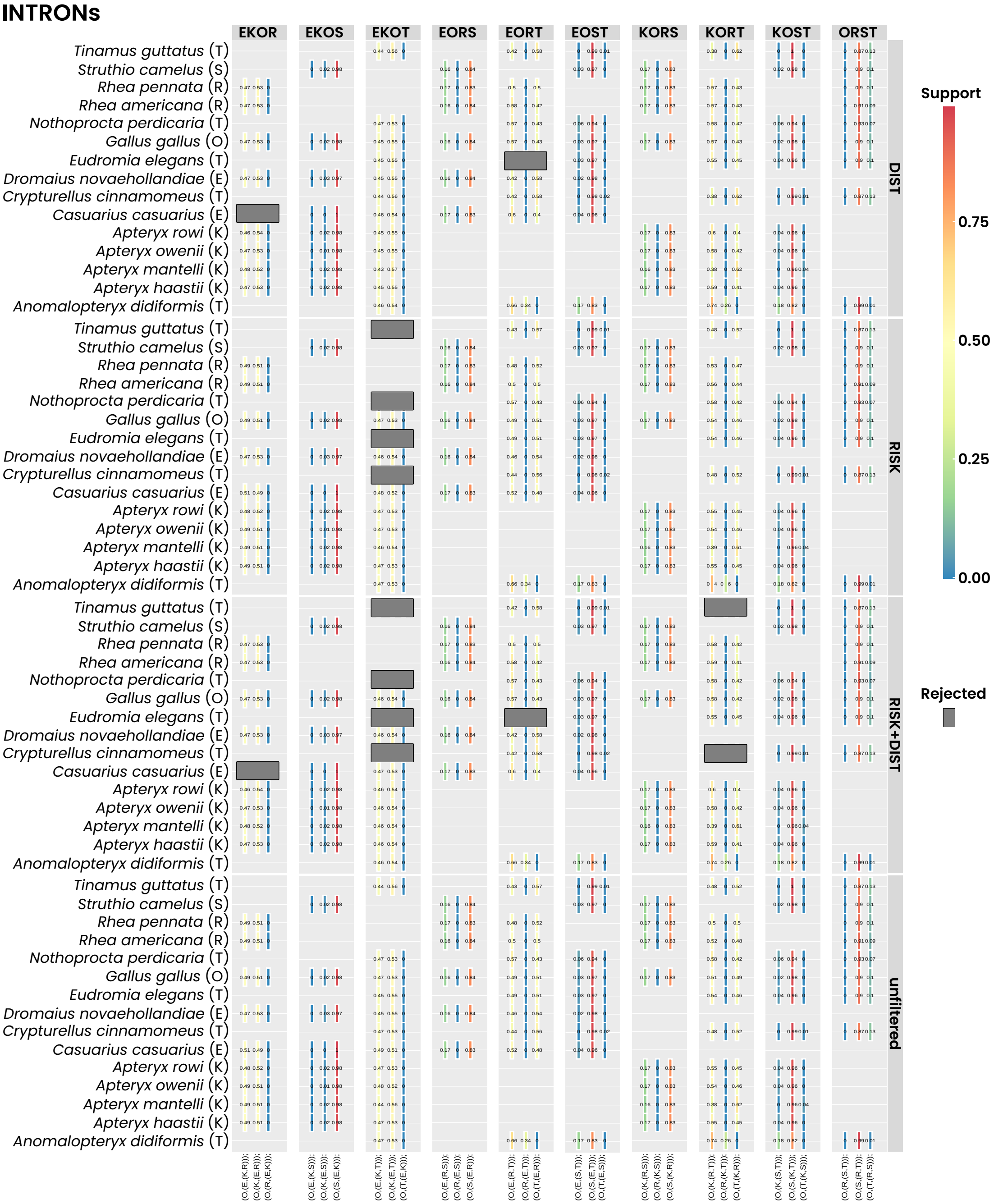

Figure 6: **Species Support Contribution in Unfiltered and Filtered INTRONs (RISK, DIST, and Combined).** The median species-specific support (y-axis, left) for each of the three possible clade-quartet trees (x-axis, bottom) is shown across all ten clade-quartet combinations (x-axis, top) for unfiltered and RISK and/or DIST filtered datasets (y-axis, right). The color scale (legend, right) represents the level of support, ranging from zero (blue) to full support (red), with yellow indicating strong conflict between trees. Clade-quartet trees are highlighted according to the predominance of species support strength: red box frames indicate strong and consistent support within single clade-quartets, while yellow box frames mark conflicted support. Species contributions that were completely rejected in single clade-quartets are highlighted with gray boxes.

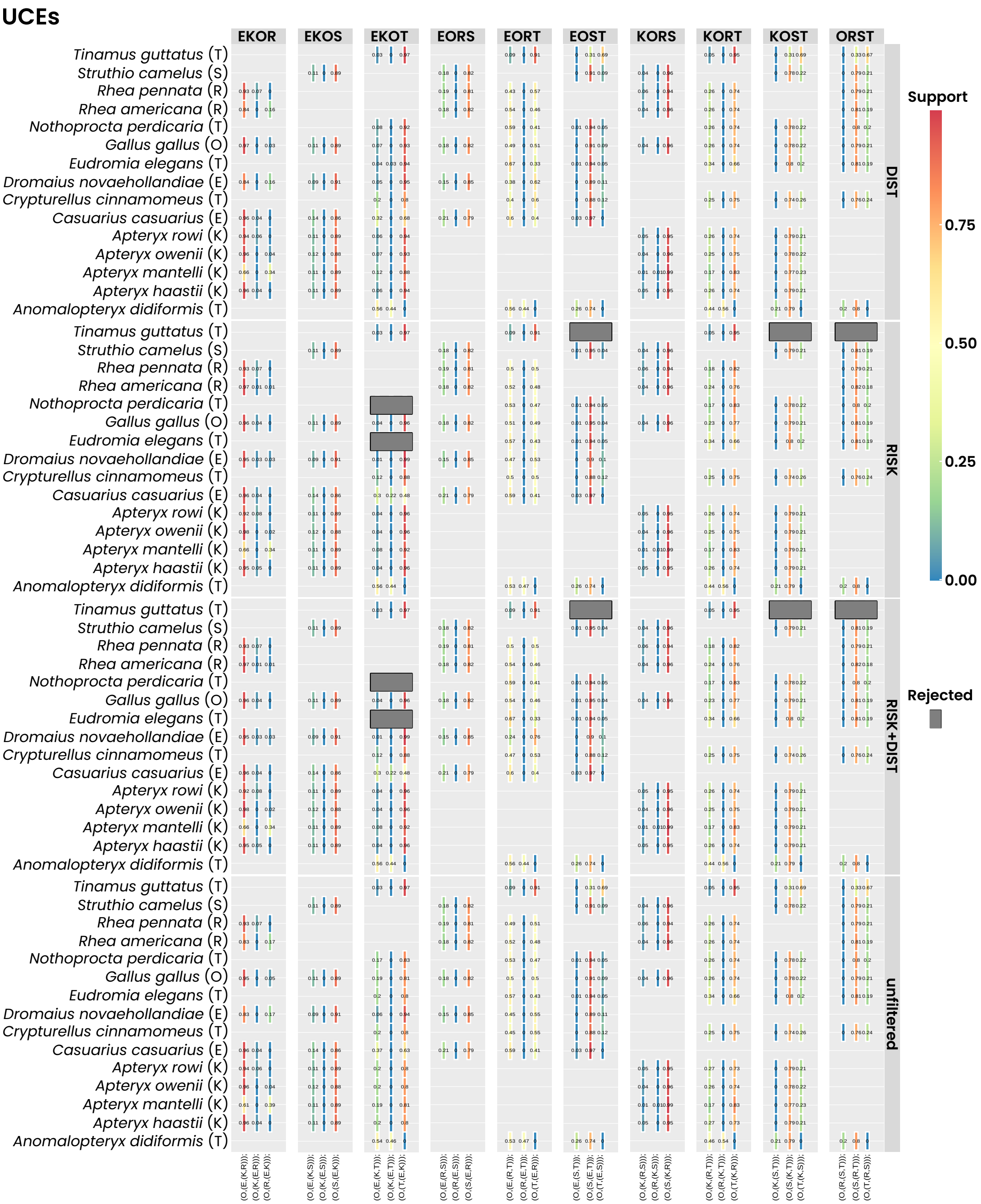

Figure 7: **Species Support Contribution in Unfiltered and Filtered UCEs (RISK, DIST, and Combined).** The median species-specific support (y-axis, left) for each of the three possible clade-quartet trees (x-axis, bottom) is shown across all ten clade-quartet combinations (x-axis, top) for unfiltered and RISK and/or DIST filtered datasets (y-axis, right). The color scale (legend, right) represents the level of support, ranging from zero (blue) to full support (red), with yellow indicating strong conflict between trees. Clade-quartet trees are highlighted according to the predominance of species support strength: red box frames indicate strong and consistent support within single clade-quartets, while yellow box frames mark conflicted support. Species contributions that were completely rejected in single clade-quartets are highlighted with gray boxes.

1.3 Maximum-Likelihood (ML) Tree Comparison

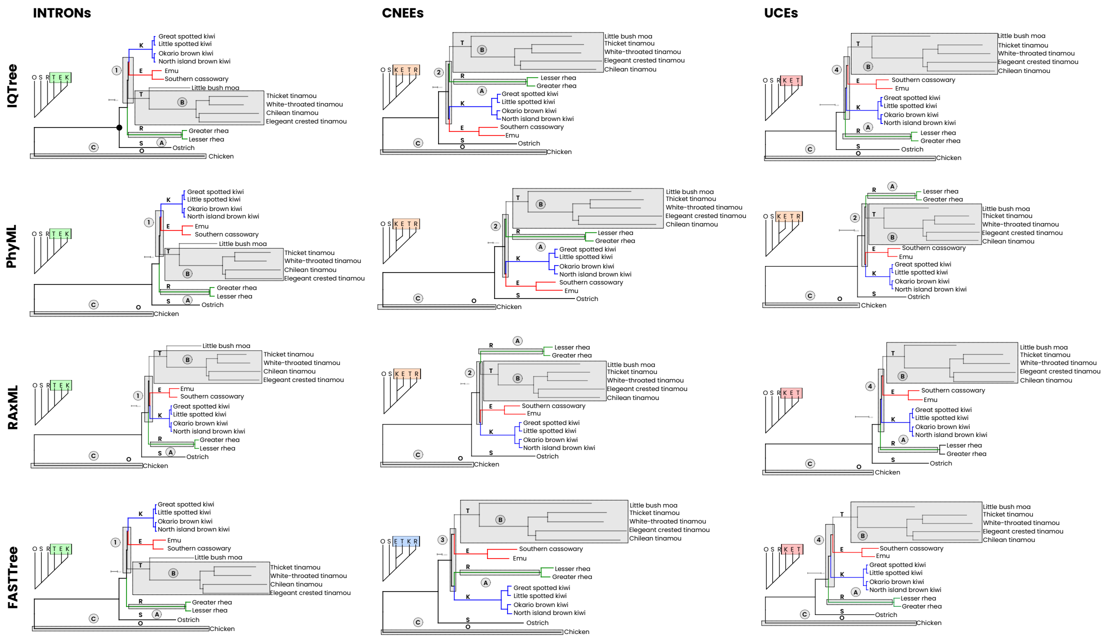

Figure 8: **Maximum-Likelihood (ML) Best Estimated Trees and Difficult-to-Resolve Relationships.** ML trees inferred using four different ML methods (y-axis) across the three datasets (x-axis, top). Identical non-Ostrich branch relationships inferred by different ML methods are highlighted with matching colored boxes. All four ML methods consistently reveal significant branch length heterogeneity, particularly for very short internal branches (1) that connect much longer clade branches (Rhea: A; Tinamou: B) with moderately long branches (Kiwi: blue; Emu: red). The single outgroup branch (C) remains distinctly separated from the ingroup, further illustrating the challenges in resolving these relationships.
